# The Shine-Dalgarno sequence of riboswitch-regulated single mRNAs shows ligand-dependent accessibility bursts

**DOI:** 10.1038/ncomms9976

**Published:** 2016-01-19

**Authors:** Arlie J. Rinaldi, Paul E. Lund, Mario R. Blanco, Nils G. Walter

**Affiliations:** 1 Single Molecule Analysis Group, Department of Chemistry, University of Michigan, Ann Arbor, Michigan 48109, USA; 2 Program in Chemical Biology, University of Michigan, Ann Arbor, Michigan 48109, USA

## Abstract

In response to intracellular signals in Gram-negative bacteria, translational riboswitches—commonly embedded in messenger RNAs (mRNAs)—regulate gene expression through inhibition of translation initiation. It is generally thought that this regulation originates from occlusion of the Shine-Dalgarno (SD) sequence upon ligand binding; however, little direct evidence exists. Here we develop Single Molecule Kinetic Analysis of RNA Transient Structure (SiM-KARTS) to investigate the ligand-dependent accessibility of the SD sequence of an mRNA hosting the 7-aminomethyl-7-deazaguanine (preQ_1_)-sensing riboswitch. Spike train analysis reveals that individual mRNA molecules alternate between two conformational states, distinguished by ‘bursts' of probe binding associated with increased SD sequence accessibility. Addition of preQ_1_ decreases the lifetime of the SD's high-accessibility (bursting) state and prolongs the time between bursts. In addition, ligand-jump experiments reveal imperfect riboswitching of single mRNA molecules. Such complex ligand sensing by individual mRNA molecules rationalizes the nuanced ligand response observed during bulk mRNA translation.

## Abstract (web summary)

In response to intracellular signals, bacterial translational riboswitches embedded in mRNAs can regulate gene expression through inhibition of translation initiation. Here, the authors describe SiM-KARTS, a novel approach for detecting changes in the structure of single RNA molecules in response to a ligand.

## Graphical abstract

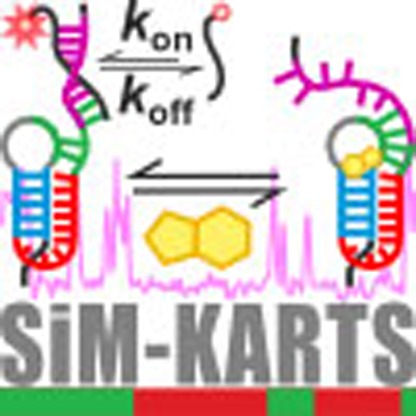

Riboswitches are noncoding structural elements most commonly embedded in the 5′ untranslated region (UTR) of bacterial messenger RNAs (mRNAs) that regulate the expression of a downstream gene through the binding of an intracellular signal^1^,^2^,^3^,^4^,^5^. These signals include nucleobases^6^,^7^, amino acids^8^,^9^, cofactors of metabolic enzymes^10^,^11^ and metal ions^12^,^13^,^14^, among others. Genetic regulation is achieved through a multitude of mechanisms; however, the two most common modes are transcriptional attenuation and inhibition of translation initiation. In translational riboswitches, ligand binding sequesters the Shine-Dalgarno (SD) sequence of the mRNA through alternative base pairing, resulting in occlusion of the ribosomal binding site needed for efficient initiation of translation. In recent years, a plethora of biophysical techniques has been employed to understand the genetic regulation mechanism utilized by translational riboswitches^15^,^16^,^17^,^18^,^19^,^20^,^21^; however, these techniques have rarely involved the entire mRNA and have largely failed to provide direct mechanistic insight at a molecular level into the coupling of ligand-induced conformational changes with downstream regulatory effects.

The preQ_1_ riboswitch from *Thermoanaerobacter tengcongensis* (*Tte*) is a strikingly small translational riboswitch that responds to the presence of the modified nucleobase preQ_1_ (ref. 22). Crystal structures^18^, in combination with single-molecule fluorescence resonance energy transfer (smFRET), NMR and computational studies^19^, have indicated that this riboswitch achieves genetic regulation by the formation of a pseudoknot, in which the P2 helix containing the first two nucleotides of the SD sequence is formed. In the absence of ligand, this helix is only transiently closed^19^, presumably revealing the SD sequence in its entirety as part of the downstream expression platform to bind the anti-SD sequence of the 16S ribosomal RNA (rRNA, Fig. 1a,b). These and many similar studies^21^,^22^ have provided valuable insight into the conformational sampling of the preQ_1_ riboswitch as well as other translational riboswitches as a function of ligand concentration. They have left largely unresolved, however, the molecular mechanism by which sequestration of just a small fraction of the SD sequence brings about the proposed ON/OFF gene-regulatory control of an entire mRNA through coupling between the aptamer and expression platform.

We here develop a technique termed Single Molecule Kinetic Analysis of RNA Transient Structure (SiM-KARTS), wherein a short, fluorescently labelled nucleic acid probe, whose sequence is complementary to a particular region of interest, is used to probe changes in structure of a longer target RNA through repeated binding and dissociation events. In the current implementation, an RNA probe corresponding to the 3′ sequence of *T. tengcongensis* (*Tte*) 16S rRNA (that is, an anti-SD probe) binds to preQ_1_ riboswitch-containing, surface-immobilized, single *Tte* mRNA molecules, and thus directly reports on the accessibility of the SD sequence. Our results reveal unexpected complexities of ligand-induced riboswitching during translation initiation and add a new dimension to an emerging model, wherein stochastic single-molecule events contribute to fine-tuned temporal gene expression fluctuations in bacteria. We anticipate that SiM-KARTS will find broad application in probing dynamic RNA structural elements at the single-molecule level.

## Results

### preQ_1_ regulates translation of the *Tte* mRNA *in vitro*

**Figure 1:**
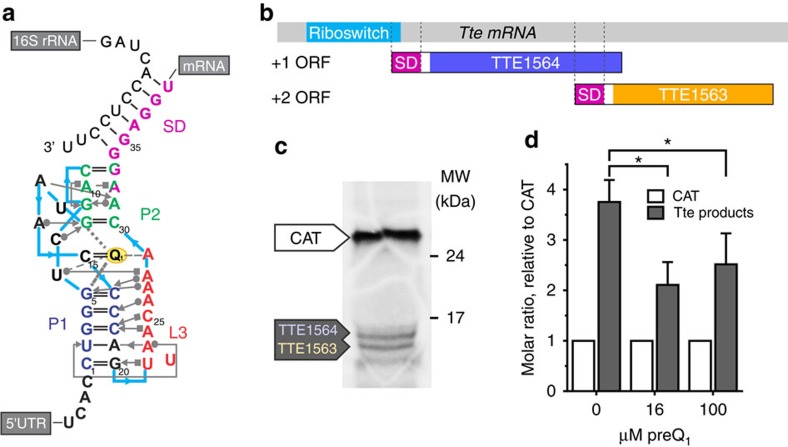
*In vitro* translation of *Tte* mRNA. (**a**) Structural map of the *Tte* preQ_1_ translational riboswitch displayed with Leontis–Westhof notations^57^. The SD sequence (purple) partially overlaps the P2 stem nucleotides (green). Formation of the P2 stem interferes with proper base pairing between the anti-SD sequence at the 3′ end of the 16S rRNA and the SD sequence of the *Tte* mRNA. (**b**) Schematic of *Tte* mRNA used for *in vitro* translation assays. The putative mRNA transcript is bicistronic, containing the overlapping reading frames for TTE1564 and TTE1563. The preQ_1_ riboswitch aptamer (light blue) overlaps with a portion of the SD sequence (purple) of TTE1564. (**c**) Example autoradiograph of *in vitro* translation products. *Tte* mRNA was translated using L-[^35^S]-Cys in the presence of the control mRNA encoding CAT at a 4:1 ratio of *Tte*:CAT mRNA. Molecular weight markers are indicated on the right (full-length gel is shown in Supplementary Fig. 11). (**d**) Quantification of *in vitro* translation products as a function of preQ_1_ concentration. The total protein produced from the *Tte* mRNA (sum of TTE1564 and TTE1563 bands, grey bar) is reported relative to the intensity of the CAT product in the same lane, after normalizing for the cysteine content of each protein (5, 1 and 1 for CAT, TTE1564 and TTE1563, respectively). The results represent the mean±s.d. of three replicates (*P<0.05).

The expression platform of translational riboswitches contains the SD sequence, a short (three to eight nucleotides, nt), purine-rich sequence located ∼5–9 nt upstream of the start codon of bacterial mRNAs^23^. It hybridizes with the 3′ end of 16S rRNA (Fig. 1b), and this interaction is important for canonical initiation and proper start codon selection by the 30S ribosomal subunit^24^. To date, a majority of riboswitch studies have focused on the properties of only the isolated aptamer domain^21^. Studies incorporating the full riboswitch including the downstream expression platform have mostly involved reporter gene assays replacing the mRNA's native gene(s) with the exogenous reporter. Here we instead opted to test the functional activity of the preQ_1_ riboswitch in the context of its native mRNA. In *T. tengcongensis*, the riboswitch is located in the 5′ UTR of a putative bicistronic operon as the *Tte* mRNA encodes two genes (Fig. 1b). *In vitro* translation using purified *Escherichia coli* translation factors and ribosomes, which share the anti-SD sequence of *T. tengcongensis* 16S rRNA with the exception of an additional 3′ single-nucleotide overhang (Supplementary Fig. 1a), produced the corresponding two proteins, TTE_RS07450 and TTE_RS07445 (subsequently referred to by their former locus tags TTE1564 and TTE1563, respectively), as expected (Fig. 1c and Supplementary Fig. 1b,c). We next performed competition experiments using a 4:1 molar ratio of *Tte* to chloramphenicol acetyltransferase (CAT) control mRNA, where the latter encodes CAT, does not contain the preQ_1_ riboswitch and thus is not expected to be modulated in its translation by preQ_1_ (Supplementary Fig. 2a). We observed an mRNA-specific, ∼40% decrease in translation of the two *Tte* mRNA genes on addition of saturating concentrations (16 and 100 μM, see below) of preQ_1_ (Fig. 1d; note that the quantification accounts for the higher number of labelled cysteines in CAT, see Methods). This result suggests that preQ_1_ decreases the translational efficiency of *Tte* mRNA, and that the native mRNA is thus responsive to ligand-induced structural changes.

### SiM-KARTS detects changes in the structure of single mRNAs

**Figure 2:**
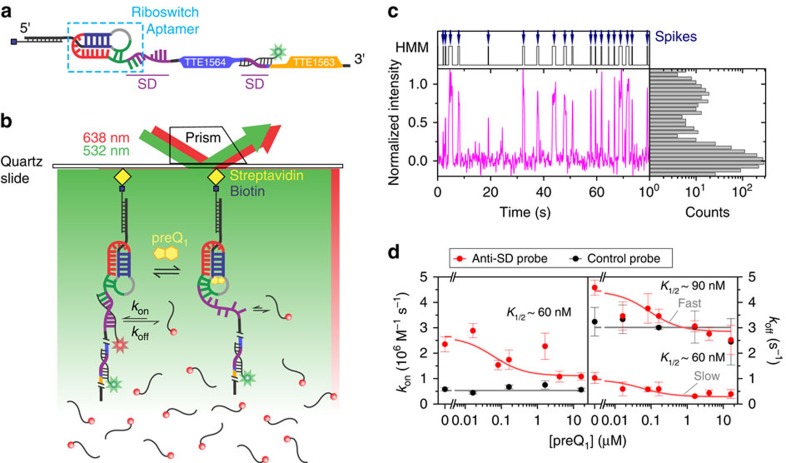
SiM-KARTS measurements of preQ_1_-dependent anti-SD-binding kinetics. (**a**) The *Tte* mRNA complex used in SiM-KARTS experiments. Full-length *Tte* mRNA molecules are immobilized to the slide surface via a biotinylated-capture strand that is hybridized to the 5′ end of the mRNA. Features of the riboswitch and associated reading frames are coloured as in Fig. 1a,b, respectively. A TYE563-LNA (with green star) is hybridized to the start of the downstream open reading frame to occlude this second SD sequence and to locate mRNAs on the slide surface. (**b**) Experimental prism-based TIRFM set-up. The *Tte* mRNA complexes shown in **a** are immobilized to a slide surface that has been passivated with biotinylated BSA (omitted for clarity). Repeated binding and dissociation of the anti-SD probe labelled with Cy5 (red sphere, red star) is monitored through co-localization of TYE563 and Cy5 fluorescence. (**c**) Representative anti-SD probe-binding fluorescence versus time trajectory and corresponding fluorescence intensity histogram for a single *Tte* mRNA molecule in the absence of preQ_1_. Cy5 intensity from the anti-SD probe (magenta) and Hidden Markov idealization to a two-state model (HMM, grey) are plotted as a function of time. The TYE563 fluorescence trace used to identify and localize the *Tte* mRNA has been omitted for clarity. (**d**) Anti-SD (red) and control (black) probe binding and dissociation rate constants (*k*_on_, left plot; *k*_off_, right plot) were determined from exponential fits of dwell times in the unbound and bound states, respectively, as a function of preQ_1_ concentration. Binding and dissociation rate constants for the control probe are unaffected by preQ_1_ concentration. The corresponding *K*_1/2_ value from the saturation curve fit of the anti-SD probe binding is indicated. The results represent the average±s.e.m. of at least three independent experiments.

To observe changes in SD sequence accessibility as a function of ligand concentration, we developed SiM-KARTS (Fig. 2), utilizing a short, fluorescently (Cy5) labelled RNA anti-SD probe with the sequence of the 12 nt at the very 3′ end of *T. tengcongensis* 16S rRNA (Fig. 1a and Supplementary Note 1). Target mRNA molecules were hybridized with a high-melting-temperature TYE563-labelled locked nucleic acid (TYE563-LNA) for visualization, immobilized on a quartz slide at low density via a biotinylated capture strand and imaged with single-molecule sensitivity by total internal reflection fluorescence microscopy (TIRFM, Fig. 2a,b). To simplify our analysis in the context of the full-length mRNA, we chose the TYE563-LNA marker to also block the distinct SD sequence and start codon of the TTE1563 open reading frame (ORF), preventing the anti-SD probe from binding to the downstream TTE1563 SD (Fig. 2a and Supplementary Fig. 3). TYE563 fluorescence could only be observed once all three components (biotinylated capture strand, *Tte* mRNA and TYE563-LNA) were assembled on the surface (Fig. 2b and Supplementary Fig. 4), attesting to the high specificity of the experiment.

Because the interaction between the *Tte* mRNA and the anti-SD probe is limited to seven Watson–Crick base pairs and one wobble (Fig. 1a), binding of the probe to a single mRNA molecule under equilibrium conditions is reversible and transient (Fig. 2b). In addition, since the experiment is performed using TIRFM, only probe molecules transiently immobilized to the slide surface via the mRNA target will be observed within the evanescent field and co-localized with TYE563 in a diffraction-limited spot, whereas probes diffusing freely in solution will instead contribute to a modest background fluorescence. Repeated, transient diffraction-limited co-localization of Cy5 and TYE563 fluorescence therefore unambiguously characterizes individual binding events of the anti-SD probe to a single target mRNA molecule (Fig. 2b). Since changes in the probe binding and dissociation time constants can be sensitively monitored over an arbitrarily long time window with high precision, these characteristic repeat signals are expected to quantitatively report on the accessibility of the SD sequence and thus secondary structure of individual mRNA molecules.

Previous SAXS^18^, smFRET and MD simulation data^19^ on the *Tte* preQ_1_ riboswitch aptamer have found that helix P2 of the riboswitch pseudoknot is partially open in the absence of preQ_1_, leaving the SD sequence more exposed than it is in the presence of ligand (Fig. 1a). To ask whether SiM-KARTS detects the expected difference in accessibility of the SD sequence under equilibrium conditions in the absence and presence of preQ_1_, Cy5-labelled anti-SD probe was flowed on a slide with immobilized and TYE563-LNA-bound *Tte* mRNA first in the absence of ligand. Demonstrating the highly parallel nature of SiM-KARTS, thousands of transient binding events were observed in over 100 mRNA molecules per experiment. The resulting Cy5 emission trajectories were fit using a two-state Hidden Markov Model (HMM) to extract dwell times of the probe in the bound and unbound states, *τ*_bound_ and *τ*_unbound_, respectively (Fig. 2c). The use of HMMs allows us to objectively identify binding events in single-molecule fluorescence time trajectories that are inherently noisy because of the background of excess free Cy5-labelled probe in solution. HMMs filter out this noise and can detect binding events as short as a single camera integration time. HMMs also offer an advantage over simple signal thresholding or image spot finding algorithms because HMMs inherently consider the kinetics of binding events that are the key information sought from SiM-KARTS. Using HMM analysis, we found that a lower-than-average signal-to-noise ratio of some trajectories sometimes could lead to misidentification of the molecule's state (bound or unbound). To further improve the HMM analysis, refitting was performed on select trajectories where binding events were clearly missed (Methods). Although this additional refitting improved the fits of a small number of individual trajectories, the rate constants were only modestly affected (Supplementary Fig. 5). This suggests that the analysis of SiM-KARTS data is robust, provided sufficient numbers of molecules are sampled (Supplementary Note 2). The resulting cumulative *τ*_bound_ and *τ*_unbound_ dwell time distributions were fit with a single-exponential function to calculate the binding rate constant *k*_on_ and a double-exponential function to extract a fast and a slow dissociation rate constant *k*_off_, respectively, based on an analysis of the residuals (Supplementary Fig. 6). In the absence of ligand, the anti-SD probe binds with a bimolecular rate constant *k*_on_ of 2.4±0.3 × 10^6^ M^−1^ s^−1^ and dissociates with two unimolecular rate constants *k*_off_ of 4.6±0.2 s^−1^ (relative amplitude=76%) and 1.0±0.2 s^−1^ (relative amplitude=24%; Fig. 2d and Supplementary Table 1 and Supplementary Note 3). As our further analysis will demonstrate, the apparent biphasic nature of *k*_off_ is simply the result of the shortcomings of a phenomenological fit to a set of complex, kinetically broadly distributed molecular behaviours.

Next, mRNA molecules were folded in the presence of varying concentrations of preQ_1_ and subjected to SiM-KARTS. The value of *k*_on_ of the anti-SD probe decreased as the concentration of preQ_1_ increased, with a half-saturation point *K*_1/2_ of ∼60 nM preQ_1_ (Fig. 2d). Such a decrease in the binding rate indicates an occlusion of this target sequence in the presence of preQ_1_, as expected. It is important to note that this *K*_1/2_ is related to, but not a direct measure of, ligand affinity, which is known to be in the low nanomolar range^18^. Instead, it reflects preQ_1_-induced structural changes in the expression platform, in contrast to previous studies performed on only the minimal aptamer^19^. Unexpectedly, increasing preQ_1_ concentrations also resulted in a decrease in both the fast and slow *k*_off_ rate constants (Fig. 2d), indicating that high preQ_1_ concentrations stabilize the SD:anti-SD interaction once formed. A plausible explanation for such an effect is the potential for preQ_1_ to stabilize co-axial stacking of the anti-SD probe on a more fully formed P2 helix (Fig. 1a).

To test whether these changes are indeed due to conformational rearrangements near the SD sequence of the riboswitch, we performed equilibrium SiM-KARTS using a Cy3-labelled blocking strand that anneals to a 35-nt region, effectively sequestering the expression platform as well as the initial five nucleotides in the TTE1564 ORF and disrupting the riboswitch aptamer domain (Supplementary Fig. 7a and Supplementary Note 1). Performing equilibrium SiM-KARTS experiments in the presence of this blocking strand along with the TYE563-LNA strand results in a situation where both SD sequences are occluded from anti-SD probe binding. Accordingly, the probe dissociation constant, *K*_d,effective_, for the mRNA at high ligand concentration is significantly lower than that for mRNA in the absence of ligand but annealed with the blocking strand (Supplementary Fig. 7e, red bar versus red striped bar). This indicates that the affinity of the anti-SD probe for the *Tte* mRNA is greatly reduced when the expression platform of the riboswitch is blocked. In addition, while the *k*_on_ of the anti-SD probe in the presence of the blocking strand (0.82 × 10^6^ M^−1^ s^−1^, Supplementary Table 2) is comparable to that of the probe in the presence of high ligand concentration (1.1 × 10^6^ M^−1^ s^−1^, Supplementary Table 1), the values of *k*_off_ differ substantially (8.3 versus 2.1 s^−1^, respectively), indicating that any spurious, nonspecific events are characterized by significantly faster probe dissociation (Supplementary Fig. 8b). This suggests that, while both conditions (blocking strand or high ligand) induce a conformation in which the SD sequence is (partially) blocked, the ligand induces a distinct kinetic profile. Conservatively assuming that the entire bound dwell time distribution for *Tte* mRNA annealed with the blocking strand represents nonspecific probe-binding events, we can proportionally subtract this background from the distribution in the presence of high ligand to estimate that a majority (>60%) of all binding events must be specific to the SD sequence under these conditions (see Supplementary Note 1 and Supplementary Fig. 8c). This unequivocally demonstrates that most binding events we observe in our equilibrium SiM-KARTS experiments are localized at the upstream SD sequence.

Finally, to further confirm the specificity of our assay, we performed equilibrium SiM-KARTS experiments using a control probe complementary to a region distal to the riboswitch and within the open reading frame of the mRNA (Supplementary Fig. 3 and Supplementary Note 4), whose structure and, thus, accessibility to control probe binding should be unaffected by preQ_1_. Indeed, the kinetics of the binding of this probe showed little change in response to preQ_1_ concentration (Fig. 2d), indicating that the ligand-induced conformational changes are localized to the SD region of the riboswitch.

### Periods of high SD sequence accessibility occur in bursts

**Figure 3:**
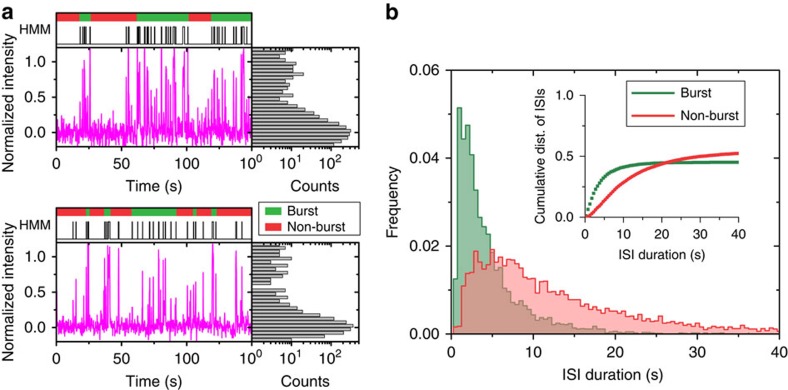
Detection of burst behaviour through spike train analysis. (**a**) Representative trajectories as in Fig. 2b for two single *Tte* mRNA molecules in the absence of preQ_1_, annotated with bursts (green bars) and non-burst periods (red bars) detected through spike train analysis. (**b**) Cumulative histogram displaying the distribution of ISIs during burst (green) and non-burst (red) periods in the absence of preQ_1_.

Further inspection of individual probe-binding trajectories revealed that single molecules interconvert between periods of frequent probe-binding events and periods of more sporadic events, which can be interpreted as periods of high and low SD accessibility, respectively (Fig. 3a). Traditional analysis methods of single molecules in aggregate failed to detect these changes. For example, common scatter plots of the mean *τ*_bound_ and *τ*_unbound_ dwell times for individual molecules^25^,^26^,^27^,^28^,^29^,^30^ in the presence of saturating ligand concentration revealed a shift towards longer unbound times compared with the absence of ligand; however, all molecules generally fit within a single broad distribution (Supplementary Fig. 8a). This observation suggests that calculating average rate constants for each mRNA molecule, while revealing heterogeneity among a population of molecules^25^,^26^,^27^,^28^,^29^,^30^, largely fails to detect time evolution in the equilibrium behaviour of a single molecule.

**Figure 4:**
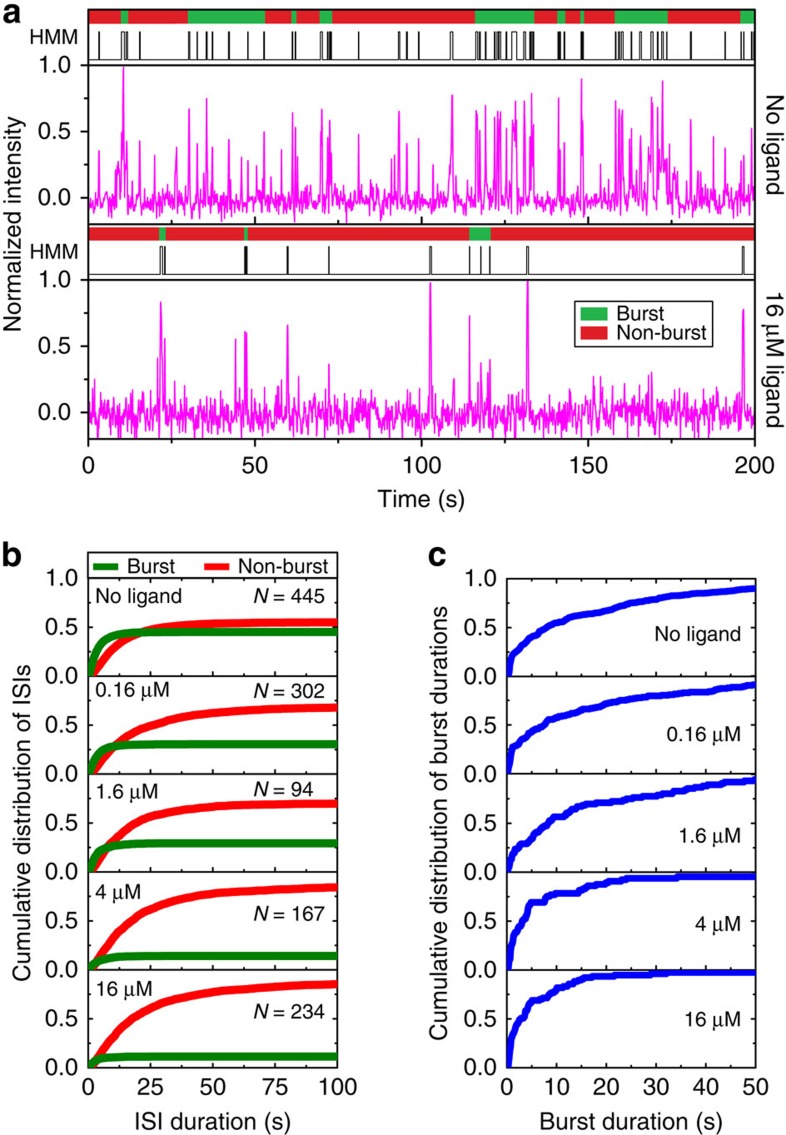
Ligand-dependent changes in bursting behaviour of single riboswitches. (**a**) Single-molecule trajectories as in Fig. 3a but in the absence and presence of saturating preQ_1_ (top and bottom, respectively). (**b**) Cumulative distribution plots indicating the distribution of ISIs during burst (green) and non-burst (red) periods at varying preQ_1_ concentrations, where *N* is number of molecules included in the analysis. (**c**) Cumulative distribution plots of burst duration for the molecules in **b** as a function of preQ_1_ concentration. As preQ_1_ concentration increases, the average burst duration decreases.

The probe-binding events detected via SiM-KARTS strongly resemble neuronal spike trains, where neuronal firing is monitored and detected as sharp, transient increases (or ‘spikes') in electrical activity in response to external stimuli. A common feature of these spike trains is short intervals of high firing activity, or ‘bursts', separated by periods of relative inactivity (non-bursts)^31^. This type of analysis previously has been used to describe transcription time series in *E. coli*^32^,^33^, leading us to ask whether spike train analysis could detect and separate in an unbiased manner the periods of high and low frequency of probe-binding events observed within single molecules. To specifically justify its use in the context of SiM-KARTS, we first calculated the Fano factor^34^ of the number of spikes within a certain time window (Supplementary Fig. 9). The Fano factor here is defined as the variance of the number of spikes within a certain time frame divided by the mean. For a random Poisson distribution, that is, one that is completely independent of the time window, the Fano factor is consistently equal to one (simulated data points in Supplementary Fig. 9). Our SiM-KARTS data clearly deviate from this expectation (Supplementary Fig. 9), rendering spike train analysis an appropriate tool. Next, exploiting its nonparametric approach we applied the Rank Surprise (RS) method of burst detection, which has been utilized to detect regions of high spike activity^31^ that, in our case, represent periods of high SD accessibility. The RS method does not make any assumptions about the distribution of spikes and is based solely on the definition of bursts as representing many spikes in a comparably short amount of time^31^. Global burst analysis across the various concentrations of ligand tested found that individual molecules displayed detectable bursts of anti-SD probe binding, which were separated by non-bursting periods characterized by areas of low average binding activity (Fig. 3a). When the duration of interspike intervals (ISIs), equivalent to *τ*_unbound_ dwell times, in the bursting and non-bursting periods were plotted, two distinct, previously hidden intramolecular behaviours became evident (Fig. 3b). We found that single molecules typically interconvert between periods of bursting and non-bursting behaviour, rather than segregating into separate subpopulations of highly and poorly accessible molecules, with bursts of high SD accessibility identified even at saturating ligand concentrations (Fig. 4a). This finding suggests that *Tte* mRNA switches between (at least) two distinct conformational states: a bursting state with overall high SD accessibility and frequent binding events of the anti-SD probe (that is, shorter ISIs) and a non-bursting state characterized by low SD accessibility where the SD sequence is more sequestered away from the probe (longer ISIs). The latter non-bursting state is adopted even in the absence of ligand (Fig. 4a), in accord with previous studies indicating that the P2 helix, which partially sequesters the SD sequence, can form without ligand present^18^,^19^.

### preQ_1_ decreases the number of bursts and the burst duration

Visual inspection of the trajectories at high ligand concentration suggested that the bursting state is shorter lived, and non-bursting periods longer lived, compared with low ligand concentration conditions (Fig. 4a). In global spike train analysis, this observation is reflected in an increasing bias towards non-burst-associated ISIs with increasing preQ_1_ concentration (Fig. 4b and Supplementary Table 1). Furthermore, the cumulative burst duration distribution shifts towards shorter values (Fig. 4c), indicating that the conformation in which the SD sequence is more accessible becomes shorter-lived and destabilized by ligand. However, the duration of ISIs, and thus the binding rate constant *k*_on_, within bursting states is largely unaffected by ligand, indicating that the bursting state conformation is similar in the absence and presence of ligand, just that its lifetime is shorter (Supplementary Fig. 10 and Supplementary Table 1). Overall, our results suggest that the *Tte* mRNA with embedded preQ_1_ riboswitch transitions between two distinguishable equilibrium conformational states: a bursting state conformation with an exposed SD sequence that is available for frequent binding of the anti-SD sequence and a non-bursting conformation with a less accessible SD sequence. Both of these states coexist and interconvert in both the presence and absence of ligand. As more ligand is added, transitions to the bursting state become less frequent and shorter lived, yet remain a persistent feature of the mRNA-embedded riboswitch.

### mRNA only partly adapts to changes in preQ_1_ concentration

**Figure 5:**
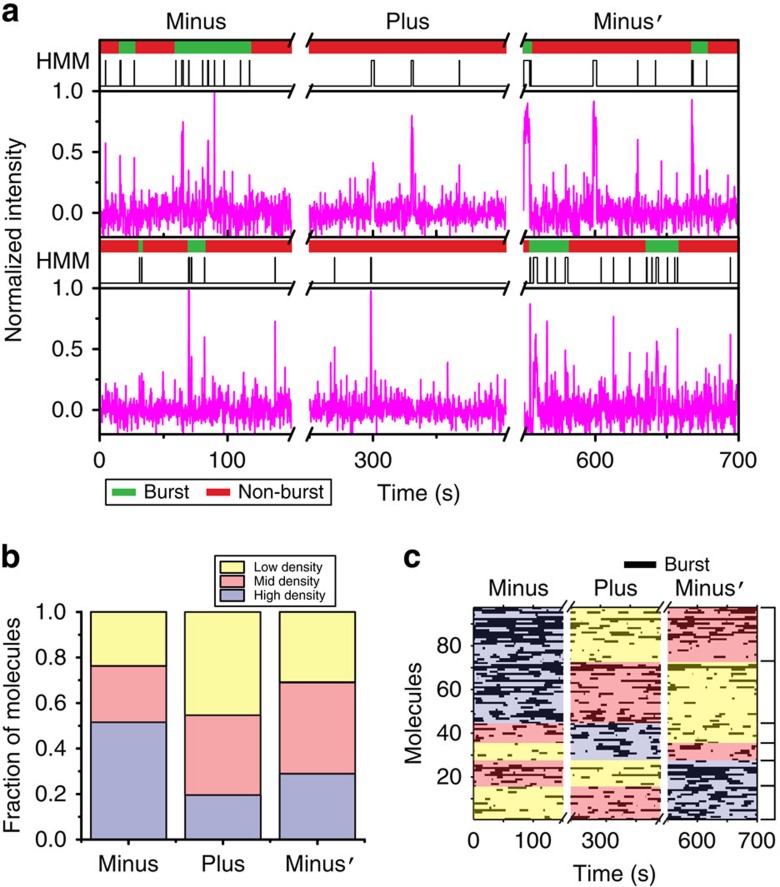
Single mRNA molecules can undergo conformational switching depending on their environment. (**a**) Exemplary single-molecule trajectories from ligand-jump SiM-KARTS experiments composed of three time segments. Anti-SD probe binding to the same set of individual *Tte* mRNA molecules is monitored first in the absence of preQ_1_ (Minus), then in the presence of 16 μM preQ_1_ (Plus) and again in the absence of preQ_1_ (Minus′). Each axis break represents a 50-s dark period between segments during which buffer was exchanged. (**b**) Distribution of burst density rankings for each segment of an individual molecule's trajectory, for all molecules in the ligand-jump SiM-KARTS experiment (*N*=97). (**c**) Rastergram displaying the bursting behaviour of the *Tte* mRNA molecules in **b**. Bursts are displayed as black bars. Individual probe-binding events (spikes) are omitted for clarity.

To assess the response of individual mRNAs and their SD accessibility to changing (nonequilibrium) ligand concentrations, as may occur in the bacterial cell, we devised a ligand-jump experiment that allowed us to apply SiM-KARTS to molecules tracked throughout a transition from no ligand, to saturating ligand and back to no ligand (Minus, Plus, Minus′ segments, respectively) in a set of contiguous fluorescence–time trajectories (Fig. 5a). We then applied global spike train analysis to all molecules we were able to track through this nonequilibrium ligand-jump experiment. To further characterize the evolution of the SD accessibility through the changes in ligand concentration, each of the three segments for a given molecule was ranked by time spent in the bursting state. An individual molecule's Minus, Plus or Minus′ segment with the highest density of bursts was ranked as High (H), the next highest as Mid (M) and the lowest as Low (L). We then quantified the overall distribution of burst ranks in the three different segments (Fig. 5b). For the majority of molecules, the highest burst density occurred in the first segment where ligand is absent (Minus), as expected. By contrast, the saturating ligand segment (Plus) exhibited mostly Low- and Mid-burst density rankings, again as expected. Finally, the Minus′ segment, where the ligand-containing buffer had been extensively washed out, exhibited a fairly equal distribution of ranks. These measures indicate that, as an ensemble, single mRNA molecules respond to ligand concentration with the expected modulation in SD accessibility. We further plotted the bursting behaviour of each of 97 molecules as a rastergram and organized them into six groups according to their per-segment burst density ranks (Fig. 5c), several of which were of particular interest. This comparison showed that ∼24% of all molecules responded to the addition and removal of ligand with reduction and recovery of bursting behaviour (H–L–M in the Minus, Plus, Minus′ sequence; molecules 75–97 in Fig. 5c and upper trace Fig. 5a), respectively, as expected. However, ∼30% of molecules seemed to not revert to greater burst density on the timescale of the experiment after the ligand was washed out (H–M–L, molecules 46–74 in Fig. 5c). This population of mRNAs reacts to initial ligand binding by SD sequence occlusion, but appears to remain in a ligand-bound conformation even after the preQ_1_-containing buffer is removed, consistent with the known slow rate of preQ_1_ dissociation^18^. Interestingly, we also observed that ∼16% of molecules displayed the opposite behaviour, that is, displayed their highest bursting density in the Minus′ segment (L–M–H, molecules 1–16 in Fig. 5b, lower trace in Fig. 5a), after the introduction and removal of ligand. This behaviour suggests that in some cases the ligand may help promote refolding of an mRNA in which the SD sequence was occluded before the addition of ligand.

## Discussion

**Figure 6:**
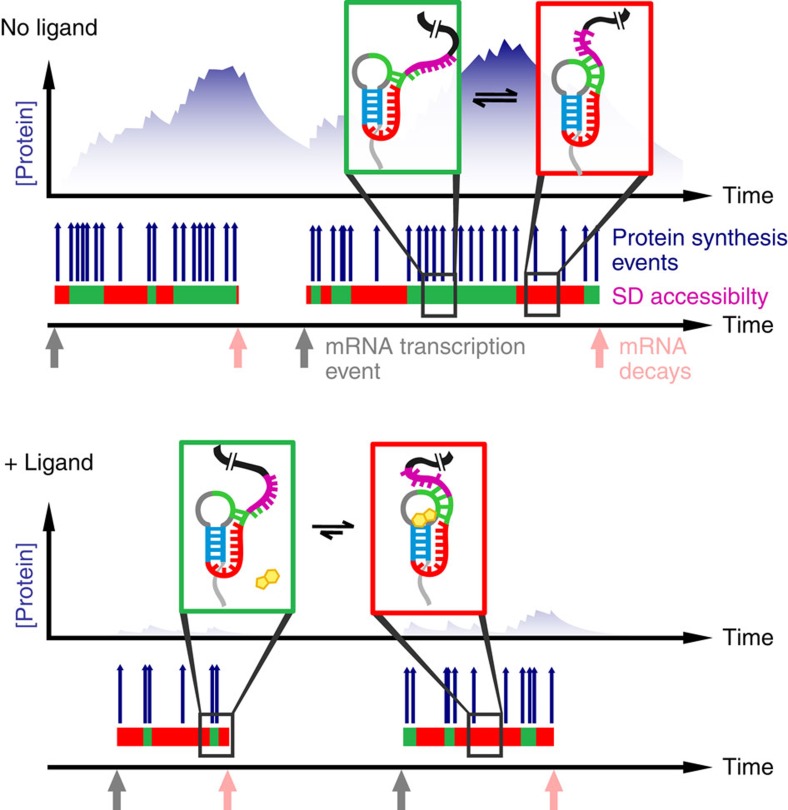
A stochastic burst model for preQ_1_-dependent expression of the *Tte* mRNA. In the absence of ligand (top), a transcription event (grey arrow) gives rise to an individual riboswitch-containing mRNA molecule that persists in the cell until it is degraded (light red arrow). During its lifetime, the mRNA transitions between burst (green bar segments) and non-burst (red bar segments) conformational states during which its SD sequence (purple nucleotides) is highly accessible and largely occluded, respectively. As reflected by increased anti-SD probe binding during SiM-KARTS, there are more potential opportunities for translation initiation events (blue arrows) in the burst state, leading to bursts of protein biosynthesis, than in the non-burst state. In the presence of the ligand (bottom), preQ_1_ favours formation of the full P2 stem (green nucleotides) and occlusion of the SD sequence, resulting in shorter excursions to the burst state and, consequently, fewer opportunities for translation initiation. This may be accentuated by a decrease in the mRNA's lifetime (depicted as overall shorter burst/non-burst bars) resulting from its scarce occupancy with actively translating ribosomes, leading to significant downregulation of protein expression. Colouring of the riboswitch cartoon is as in Figs 1a and 2a.

It is generally thought that translational riboswitches achieve gene regulation through a ligand-mediated conformational change in the aptamer domain that is then transduced into the downstream expression platform to actuate an ON/OFF switch in gene expression^1^,^2^,^3^,^4^,^5^. However, the molecular underpinnings of this transduction, especially in the context of the native gene, are still poorly studied and understood. Here we present SiM-KARTS as a cost-effective and relatively non-invasive alternative technique to FRET that sensitively probes site-specific changes in secondary structure of arbitrarily large or complex single RNA molecules in real-time and without the need for covalently modifying the targeted RNA, which can be an inefficient and expensive undertaking. Utilizing an anti-SD probe mimicking the 3′ end of the corresponding 16S rRNA, we used SiM-KARTS to quantify the accessibility of the SD sequence in the expression platform of an mRNA hosting a small preQ_1_ riboswitch over a significantly longer time window than a similar FRET experiment, since SiM-KARTS is inherently not limited by photobleaching. The resulting extended observation of single molecules is critical for long RNAs that demonstrate relatively slow changes in structure, such as the accessibility changes in the SD sequence of the mRNA studied here. As expected, we detected a decrease in SD accessibility on addition of preQ_1_ as a marked decrease in the binding rate constant *k*_on_ of the probe. Less expectedly, however, probe-binding events to a single mRNA (spikes) typically occurred in bursts. Consequently, spike train analysis of these bursts provided evidence for two conformational states repeatedly interconverting within single molecules that are characterized by periods of high and low SD accessibility. Unexpectedly, these two states were observed not only in the absence of preQ_1_, but also at saturating ligand concentration, indicating that the riboswitch continues to occasionally sample a conformation with high SD accessibility. Finally, nonequilibrium ligand-jump experiments indicated that single mRNA molecules only imperfectly switch between high and low SD accessibility. These findings rationalize the significant, but relatively modest impact of saturating preQ_1_ concentrations on the *in vitro* translation output of the mRNA and lead us to propose the model for stochastic riboswitch-controlled gene expression depicted in Fig. 6.

Notably, previous smFRET studies of the isolated *Tte* riboswitch also found two conformations populated from zero to saturating ligand concentration, identified as pre-folded and folded, that both respond to (that is, ‘sense') ligand^19^. It is tempting to speculate that these two local conformations of the aptamer give rise to the two mRNA conformations that differentially bind the anti-SD during SiM-KARTS; the pre-folded state appears to have an only partially formed P2 helix^19^ that is expected to bind the probe more readily (resulting in a burst of spikes), whereas the folded state features a fully formed P2 (ref. 19) that disfavours probe binding and thus leads to only sparse spikes (non-bursts, Fig. 6). Consistent with the relatively modest binding free energy of −11 kcal mol^−1^ available from this small-molecule ligand^19^, the mode of action of preQ_1_ then is to subtly remodel the aptamer and only modestly reduce the hosting mRNA's translation-initiation frequency, consistent with the observed reduction of *in vitro* translation product by ∼40%. This seemingly moderate (approximately twofold) change *in vitro* that can be directly attributed to the ligand suggests that regulation by preQ_1_ is more nuanced than previously appreciated and that it may be potentiated by other forces at work in the cell, including: co-transcriptional folding of the mRNA from 5′ to 3′ ends^35^,^36^, competition between ribosome-catalysed translation and RNase-mediated mRNA decay^37^,^38^ (leading to a shorter lifetime of a sparsely translated mRNA, Fig. 6) and repeated unfolding of the RNA resulting from close spacing between the transcription complex and the leading and sequentially loaded ribosomes^39^.

The spike trains detected by SiM-KARTS resemble the transcriptional bursting that has been suggested as an underlying cause of genetic ‘noise'^33^,^40^,^41^. This generally stochastic nature of biological systems results in cell-to-cell variability and has been shown to be beneficial to organisms, particularly during times of environmental stress^42^,^43^,^44^. Each time a single mRNA molecule is transcribed it gives rise to a few to tens of protein molecules^33^. We here show that translational bursting of an mRNA, which can be modulated by ligand binding to an embedded riboswitch, appears to add another layer of stochasticity to the gene expression cascade (Fig. 6). We anticipate that such translational bursting will turn out to be a widespread phenomenon among mRNAs that generally present structurally dynamic ribosome substrates whose SD region is known to significantly influence translation efficiency^45^, and that our SiM-KARTS approach can detect changes in the secondary structure not just of single riboswitch-hosting mRNA molecules but of virtually any RNA under a wide range of conditions, poising it to find broad application.

## Methods

### Ligand and oligonucleotides

The preQ_1_ ligand used in this study was generously provided by George Garcia; preQ_1_ was synthesized following established methods^46^ and additionally purified using reverse-phase HPLC as described^47^. DNA and LNA oligonucleotides were purchased from Integrated DNA Technologies Inc. (IDT) and Exiqon, respectively. Fluorophore-labelled DNA and LNA oligonucleotides were HPLC-purified by the respective manufacturer. The control and anti-SD probe RNAs were purchased from IDT with a 5′ aminohexyl-linker modification and labelled with Cy5-NHS ester (GE Healthcare) exactly as described^19^. The sequences of all oligonucleotides and cloning primers used in this study are listed in Supplementary Note 4.

### RNA preparation

Reference genomic sequences for *T. tengcongensis* were downloaded from the National Center for Biotechnology Information (NC_003869.1, http://www.ncbi.nlm.nih.gov). The complete mRNA transcript, including the TTE_RS07450 and TTE_RS07445 (TTE1564 and TTE1563, respectively) ORFs, and its 3′ UTR as predicted from the FindTerm algorithm (SoftBerry), was amplified using PCR from *T. tengcongensis* genomic DNA, which was purchased from the NITE Biological Resource Center. The amplified region was cloned into the pUC19 plasmid between the BamHI and HindIII sites with an engineered upstream T7 promoter. For use as a control for *in vitro* translation assays, an FspI site was introduced through site-directed mutagenesis^48^ into the control vector provided with the ActivePro In Vitro Translation Kit (Ambion) containing the CAT gene under the control of a T7 promoter (pAMB CAT). All of the plasmids used in this study are available through Addgene (www.addgene.org).

mRNA was produced by *in vitro* transcription. The *Tte* pUC19 plasmid was linearized with HindIII (AflII or XbaI for *in vitro* translation assays; New England Biolabs) for run-off transcription. Similarly, the pAMB CAT plasmid was linearized with FspI (New England Biolabs). Transcription reactions were performed in the presence of 120 mM HEPES-KOH (pH 7.5 at 25 °C), 25 mM MgCl_2_, 2 mM spermidine, 40 mM dithiothreitol (DTT), 30 mM NTPs, 0.01% (w/v) Triton X-100, 200 nM linearized plasmid, 0.01 U μl^−1^ pyrophosphatase and 0.07 mg ml^−1^ T7 RNA polymerase in a total volume of 1 ml. Transcription reactions were incubated at 37 °C for 4 h. Enzyme was removed using phenol/chloroform extraction, and the resulting solution was spun in an Amicon 100 MWCO spin column to reduce the volume to ∼100 μl. mRNA was purified by denaturing, 7 M urea, PAGE, detected using brief 254-nm ultraviolet radiation and gel-eluted overnight. mRNAs were ethanol-precipitated and resuspended in TE buffer at pH 7.0. The sequences of the *Tte* mRNAs used in this study are listed in Supplementary Note 4.

### Equilibrium SiM-KARTS

An RNA mix containing 2 nM each of *Tte* mRNA, TYE563-LNA, biotin capture strand and Cy3-blocking strand (when present, see Supplementary Fig. 7 and Supplementary Note 1) were heat-annealed at 70 °C for 2 min in the presence of SiM-KARTS buffer containing 50 mM Tris-HCl (pH 7.5 at 25 °C), 0.6 M NaCl and 20 mM MgCl_2_, and were allowed to cool to room temperature over 20 min in the presence or absence of preQ_1_. Next, the RNA mix was diluted to 40 pM in the same buffer in the presence or absence of preQ_1_, with an additional 12.5-fold excess of TYE563-LNA, biotin-capture strand and Cy3-blocking strand (when present) to ensure that the complex would stay intact during dilution. All sequences of mRNA, capture strand, TYE563-LNA, Cy3-blocking strand and Cy5 anti-SD probe can be found in Supplementary Note 4. The diluted complex was chilled on ice. The chilled solution was flowed over an assembled microfluidic channel on a quartz slide coated with biotinylated BSA and streptavidin, as previously described^49^,^50^. The chilled, 40 pM RNA complex solution (100μL) was flowed over the slide and allowed to equilibrate for 5 min. Excess RNA was washed off the slide with SiM-KARTS buffer with or without preQ_1_. An oxygen-scavenging system consisting of 5 mM protocatechuic acid and 50 nM protocatechuate-3,4-dioxygenase with or without preQ_1_, to slow photobleaching, and 2 mM Trolox, to reduce photoblinking^51^, as well as 50 nM Cy5 probe was flowed over the slide and allowed to equilibrate for 5 min. Both Cy5 and TYE563 dyes were directly and simultaneously excited using 638 nm red and 532 nm green diode lasers, respectively. Emission from both fluorophores was simultaneously recorded using an intensified charge-coupled device camera (I-Pentamax, Princeton Instruments) at 100-ms time resolution using the Micro-Manager software (https://www.micro-manager.org/). Fluorescence time traces were extracted from the raw movie files using IDL (Research Systems) and analysed using Matlab (The MathWorks, Inc.) scripts. Genuine traces exhibiting binding were manually selected using the following criteria: a single photobleaching step of the TYE563 signal to localize the mRNA molecule on the slide surface, TYE563 fluorescence intensity of >200 intensity units and at least two Cy5 co-localization signals per trajectory corresponding to anti-SD-binding events with a signal to noise ratio of at least 3:1. Suitable traces were compiled. Hidden Markov Modeling analysis was performed on the Cy5 intensity using the segmental k-means algorithm in the QuB software suite^52^. A two-state model was used with an unbound and bound state to idealize the data (for an additional discussion of the idealization procedure, see Supplementary Note 2). Transition density plots were constructed to extract the dwell times in the bound and unbound states, as described^53^. The normalized cumulative distributions of bound dwell times were fit with a double-exponential, and unbound dwell times were fit with a single-exponential association function (see text) in OriginLab 8.5 from which on- and off-rates were calculated. Rate constants for the anti-SD probe as a function of preQ_1_ concentration were fit with a dose–response curve for an inhibitor with a standard Hill slope of −1. Linear regression of the data for the control probe in OriginLab showed the slope to not be significantly different from zero; these data were thus fit with regression lines of zero slope.

### Ligand-jump SiM-KARTS

Fluorescently (TYE563) labelled preQ_1_ riboswitches were immobilized as detailed above and first imaged in SiM-KARTS buffer without ligand. Initial co-localization of the TYE563 and Cy5 signals provided for unambiguous determination of the relative locations of single-mRNA molecules on the slide, even after TYE563 photobleaching. In some cases, the TYE563 signal persisted throughout both dark periods, further confirming that the same molecule was tracked throughout the duration of the experiment. Binding of anti-SD-labelled probes (Cy5) at these locations was continuously monitored to determine the accessibility of the SD sequence. SD accessibility was monitored for 150 s, and then a new solution of anti-SD probe in SiM-KARTS buffer was introduced in conjunction with preQ_1_ at saturating concentration (16 μM). The process was repeated but with a final SiM-KARTS buffer solution without ligand. Because the fluorescence measured is of the anti-SD probe, which is in great excess and only excited briefly while near the surface because of the TIRFM illumination conditions, we can observe binding events to the same mRNA molecule throughout the change in ligand concentrations with limited risk of photobleaching the rapidly dissociating anti-SD probe molecules.

### Ribosome preparation

Salt-washed ribosomes and separated ribosomal subunits were prepared using a previously described protocol with several modifications^54^. Briefly, *E. coli* MRE600 was grown in Luria-Bertani (LB) medium at 37 °C to an OD_600_ of 0.6–0.8, cooled at 4 °C for 45 min and then pelleted. All subsequent steps were performed on ice or at 4 °C. The cell pellet was resuspended in buffer A (20 mM Tris-HCl (pH 7.05 at 25 °C), 100 mM NH_4_Cl, 10 mM MgCl_2_, 0.5 mM EDTA and 6 mM β-mercaptoethanol), and the cells were lysed in a single pass using a M-110L Microfluidizer processor (Microfluidics). The lysate was cleared by centrifugation at 30,000*g*, and the clarified lysate was pelleted over a 35-ml sucrose cushion (1.1 M sucrose, 20 mM Tris-HCl (pH 7.05 at 25 °C), 500 mM NH_4_Cl, 10 mM MgCl_2_ and 0.5 mM EDTA) in a Beckman Ti-45 rotor for at least 16 h at 32,000 r.p.m. The pellet was washed with 1 ml of buffer B (20 mM Tris-HCl (pH 7.05 at 25 °C), 500 mM NH_4_Cl, 10 mM MgCl_2_ and 0.5 mM EDTA), resuspended in 10 ml of buffer B by gentle stirring and brought to a final volume of 35 ml in buffer B. This material was then pelleted again over a 35-ml sucrose cushion. The resulting pellet was washed with 1 ml of storage buffer (50 mM Tris-HCl (pH 7.5 at 25 °C), 70 mM NH_4_Cl, 30 mM KCl, 7 mM MgCl_2_ and 6 mM β-mercaptoethanol), resuspended in 2.5 ml storage buffer by gentle stirring and the dialysed against three changes of buffer C (50 mM Tris-HCl (pH 7.05 at 25 °C), 150 mM NH_4_Cl, 1 mM MgCl_2_ and 6 mM β-mercaptoethanol) to dissociate the subunits. A portion of the dialysed sample was adjusted to a final Mg^2+^ concentration of 7 mM, flash-frozen with liquid nitrogen in aliquots (salt-washed ribosomes) and stored at −80 °C. For the remaining dialysed sample, ∼100 A_260_ units of material was loaded on each of six 10–40% sucrose gradients in buffer C and separated by zonal centrifugation in a Beckman SW-28 Ti rotor for 18 h at 20,000 r.p.m. Gradient fractions containing 30S or 50S ribosomal subunit peaks were pooled separately and pelleted in a Beckman Ti-70 rotor for 12 h at 61,500 r.p.m. Pelleted subunits were resuspended in storage buffer and flash-frozen with liquid nitrogen in aliquots (separated subunits) and stored at −80 °C.

### *In vitro* translation assays

Salt-washed ribosomes and separated subunits were found to perform similarly (Supplementary Fig. 12), and therefore salt-washed ribosomes were used for all *in vitro* translation assays unless otherwise noted. *In vitro* transcribed mRNAs were translated using the PURExpress Δ Ribosome Kit (New England Biolabs). For each reaction, 3 μl of a 4-μM mRNA solution (4 μM CAT, 4 μM *Tte* mRNA or a mixture of 0.8 μM CAT and 3.2 μM *Tte* mRNA) was re-folded in the presence of 0, 16 or 100 μM preQ_1_ by heating to 70 °C for 2 min, followed by slow cooling to room temperature for 20 min, and then placed on ice. The remaining components required for translation were master-mixed and aliquoted to each reaction (1.5–9 μCi L-[^35^S]-Cysteine, 4 μl PURExpress Solution A, 1.2 μl Factor Mix and 6 pmol salt-washed ribosomes or separated 30S and 50S subunits), along with additional preQ_1_ required to maintain a final concentration of 0, 16 and 100 μM preQ_1_ in the final reaction volume of 12 μl. Reactions were incubated at 37 °C for 2 h, frozen on dry ice and stored at −20 °C. The following day, reactions were thawed at room temperature and 2 μl of 1 M KOH was added to quench the reaction and cleave any remaining peptide from their tRNA. Protein products were precipitated by adding 5 volumes of cold acetone and pelleted by centrifugation at 14,000*g* for 10 min. Pellets were resuspended in 20 μl of 1 × loading buffer (45 mM Tris-HCl (pH 8.45 at 25 °C), 10% (v/v) glycerol, 50 mM DTT, 1% (w/v) SDS and 0.01% (w/v) bromophenol blue) and heated at 37 °C for 45 min. Protein products were resolved on 16% Tris-tricine SDS–PAGE gels^55^ electrophoresed at 150 V for 2.5 h. Gels were then fixed for 45 min in 5% (v/v) glycerol, 40% (v/v) methanol and 10% (v/v) acetic acid and dried on a 3-mm Whatman paper using a Bio-Rad Model 583 Gel Dryer. Dried gels were imaged using a storage phosphorscreen and Typhoon 9410 Variable Mode Imager (GE Healthcare Life Sciences). Gel images were quantified using ImageQuant v5.2 (Molecular Dynamics). Unless otherwise noted, after background correction the intensities for CAT, TTE1564 and TTE1563 bands were divided by their respective number of cysteine residues (5, 1 and 1, respectively). For each lane, the intensities for TTE1564 and TTE1563 bands were summed and then divided by the intensity of the respective CAT band. Values at each preQ_1_ concentration were graphed in Prism (GraphPad Software) and an unpaired, two-tailed *t*-test was used to assess statistical significance.

### Burst analysis

Burst analysis was carried out using the RS method described by Gourévitch and co-workers^31^. We utilized a modified Matlab implementation of the RS method based on the Matlab script provided in the supplement to Gourévitch *et al.*^31^ In the standard implementation, each molecule's ISIs are ranked independently. In our implementation, termed global burst analysis, we have extended this so that each ISI detected in all our experimental conditions is ranked simultaneously; therefore, a burst is defined as a global property of the molecules and is not biased by the number of binding events of an individual molecule. Briefly, ISIs were determined by calculating the time in between individual binding events for each molecule. ISIs for all molecules were collected and used as an input for global burst analysis, using the RS method to demarcate the start and end points of bursts, that is, the first and last binding events, respectively, in a sequential series of binding events occurring in quick succession (‘burst'). Each start and end point was then reassigned to the corresponding molecule, preserving the single-molecule burst profile. Global burst analysis was carried out separately for equilibrium and ligand-jump data sets. The RS algorithm developed by Gourévitch *et al.*^31^ requires two parameters, the maximal ISI between spikes to be considered part of a burst and a RS cutoff (alpha). These were set to 40s and 3, respectively. Although the maximal ISI in a burst was set to 40 s, the distribution of ISIs we obtained after analysis is much lower than this, suggesting that we have provided enough flexibility in the algorithm to find the true distribution of ISIs in burst.

For the ligand-jump SiM-KARTS experiments, we further characterized the time evolution of SD accessibility throughout changes in ligand concentration by examining changes in the burst density for each molecule. We ranked the three segments (Minus, Plus, Minus′) for each molecule in the ligand-jump SiM-KARTS experiment by their burst density. For a given molecule, the segment with the highest density of bursts was ranked as High (H), the next highest as Mid (M) and lowest as Low (L). We then quantified the overall distribution of burst density rankings for the three segments. We plotted each individual molecule's burst behaviour as a rastergram and organized them such that their per-segment ranks were the same within a group. MATLAB scripts for global burst analysis are provided as part of the Supplementary Information.

### Fano factor calculations

Matlab scripts were written to calculate and simulate the Fano factor from our experimental data and from a simulated Poisson distribution, respectively. The Fano factor is defined as the variance in spike counts divided by the mean spike count for a given time interval, *T*. For every molecule analysed in a particular condition, a time interval of length *T* was randomly selected from the molecule's fluorescence time trace. The Fano factor was calculated for *T* equal to 5, 10, 20 and 40 s. Each time window *T* was sampled 100 times with a different random seed for each molecule to generate an average Fano factor. The average Fano factor and the s.d. for each time window are presented in Supplementary Fig. 9. For the simulations, the Matlab Poisson random number generator was utilized to generate spike counts with an average firing rate equal to the average ISI in the burst from our experimental data and equal number of samplings. 95% Confidence intervals were calculated in Matlab utilizing the function:

bounds=gaminv([.025,.975],(*n*−1)/2,2/(*n*−1)), where *n*=sample size^34^.

### Measurement of the anti-SD probe diffusion coefficient

Fluorescence correlation spectroscopy was used to determine the diffusion coefficient of the Cy5 anti-SD probe. Glass coverslips were passivated before use as follows: a coverslip was pre-wet with 50 μl of T50 buffer (Tris-HCl (pH 8.0 at 22 °C), 50 mM NaCl), dried with a gentle stream of air and then incubated with 1.0 mg ml^−1^ of biotinylated BSA for 10 min. The biotinylated BSA solution was then removed, the coverslip was washed once with 50 μl T50 buffer, dried and then incubated with 0.2 mg ml^−1^ of streptavidin for 5 min. The streptavidin solution was then removed, and the coverslip was finally washed twice with SiM-KARTS buffer. The anti-SD probe was diluted to 2.5 nM in SiM-KARTS buffer, a small aliquot was placed on the washed and dried coverslip and measurements were performed at room temperature on an Olympus IX81 inverted microscope with an ISS ALBA 5 confocal system (Champaign, IL). Ten replicate traces of 30 s each were acquired and sampled at 50 kHz. The data from each replicate were averaged and fit in PyCorrFit v0.9.1 (http://pycorrfit.craban.de/) with a correlation function equation (1) for three-dimensional free diffusion with a Gaussian laser excitation profile (elliptical), including a triplet component, where *n* is the effective number of particles in the confocal volume, *τ*_diff_ is the characteristic residence time in the confocal volume, *SP* is the structural parameter, *T* is the fraction of particles in the non-fluorescent triple state and *τ*_trip_ is the characteristic residence time in the triplet state^56^.


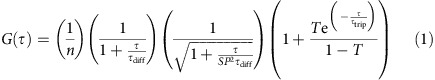


The average correlation curve is shown in Supplementary Fig. 14. A diffusion coefficient, *D*_probe_, was calculated from the fit of the average according to the relationship:


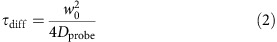


where *w*_0_ is the dimension of the Gaussian detection volume transversal to the optical axis. The uncertainty for the diffusion coefficient was estimated by re-fitting 7 randomly selected subsets of the data, where each subset contained 4 of the 10 replicates, and taking the s.d.

## Additional information

**How to cite this article:** Rinaldi, A. J. *et al.* The Shine-Dalgarno sequence of riboswitch-regulated single mRNAs shows ligand-dependent accessibility bursts. *Nat. Commun.* 7:8976 doi: 10.1038/ncomms9976 (2016).

## Supplementary Material

Supplementary Information Supplementary Figures 1-14, Supplementary Tables 1-2, Supplementary Notes 1-4 and Supplementary References
