# Supplementary material for: The Shine-Dalgarno sequence of riboswitch-regulated single mRNAs shows ligand-dependent accessibility bursts
Source: Nat Commun. 2016 Jan 19;7:8976. doi: 10.1038/ncomms9976 (PMC4735710; doi:10.1038/ncomms9976)
Supplement: Supplementary Information — Supplementary Figures 1-14, Supplementary Tables 1-2, Supplementary Notes 1-4 and Supplementary References [file ncomms9976-s1.pdf]

SUPPLEMENTARY FIGURES

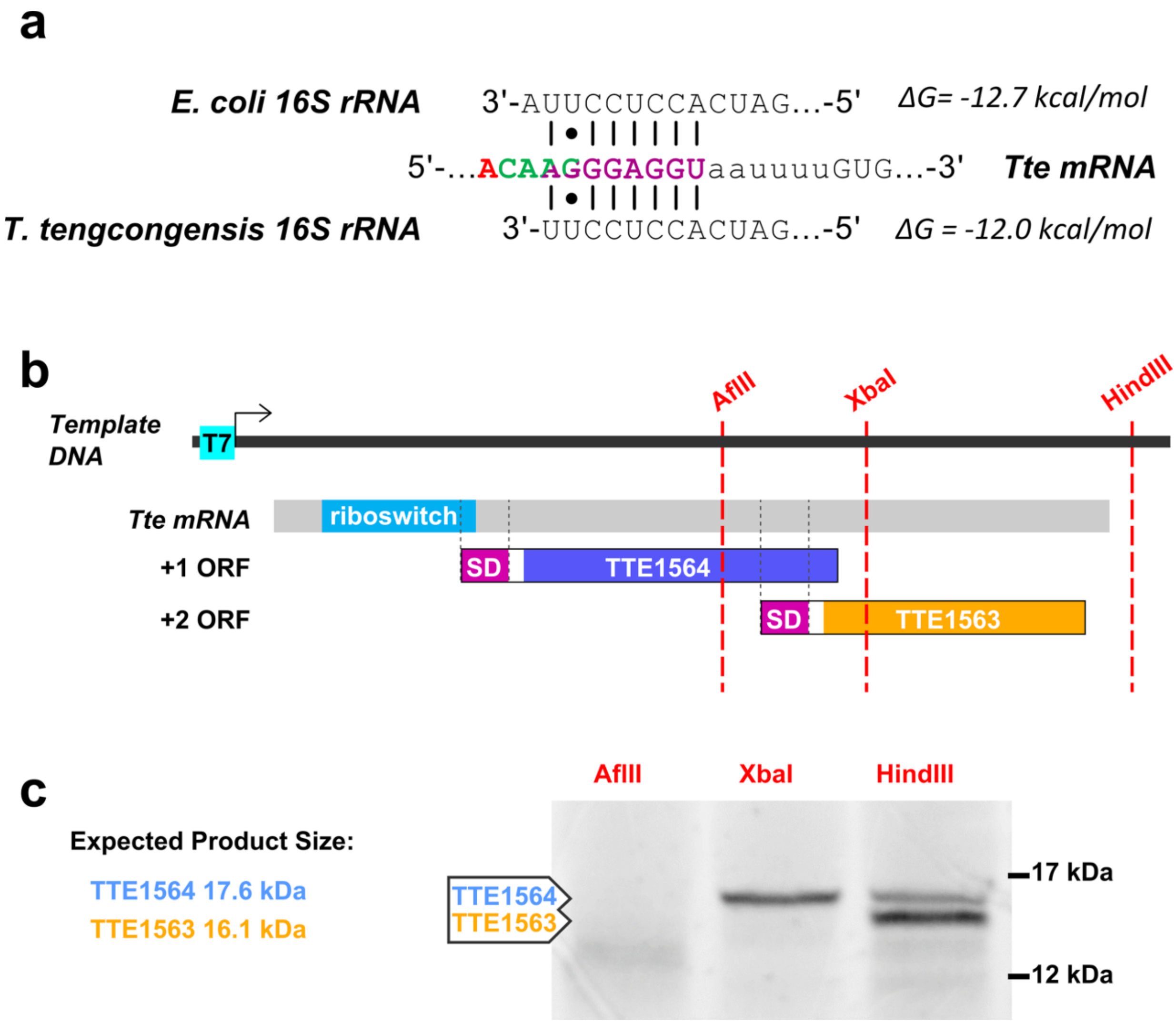

Supplementary Figure 1 | Specifics of the *in vitro* translation of *Tte* mRNA. (**a**) Comparison of Shine-Dalgarno/anti-Shine-Dalgarno pairings between the *Tte* mRNA and the 16S rRNA from *E. coli* ribosomes, used for *in vitro* translation assays, and from *T. tengcongensis*. The Shine-Dalgarno sequence (purple) partially overlaps the P2 stem nucleotides (green). The last A of L3 is shown in red. Gibbs free energies of folding were calculated in RNAstructure v5.6 (Mathews Lab). The 12 nucleotides shown for the *T. tengcongensis* 16S rRNA were used as the sequence for the anti-SD probe in SiM-KARTS experiments. (**b**) Schematic of truncated *Tte* mRNA transcripts used to positively assign bands to TTE1564 and TTE1563 products. Truncated transcripts were prepared by digesting the DNA template with specific restriction enzymes (red). Only complete ORFs with a stop codon will generate a protein product, allowing for unambiguous identification of bands. (**c**) Autoradiograph of *in vitro* translation using L-[^{35}S]-Cys of various truncations of the *Tte* mRNA. Molecular weight markers are indicated on the right (full-length gel is shown in **Supplementary Fig. 12**).

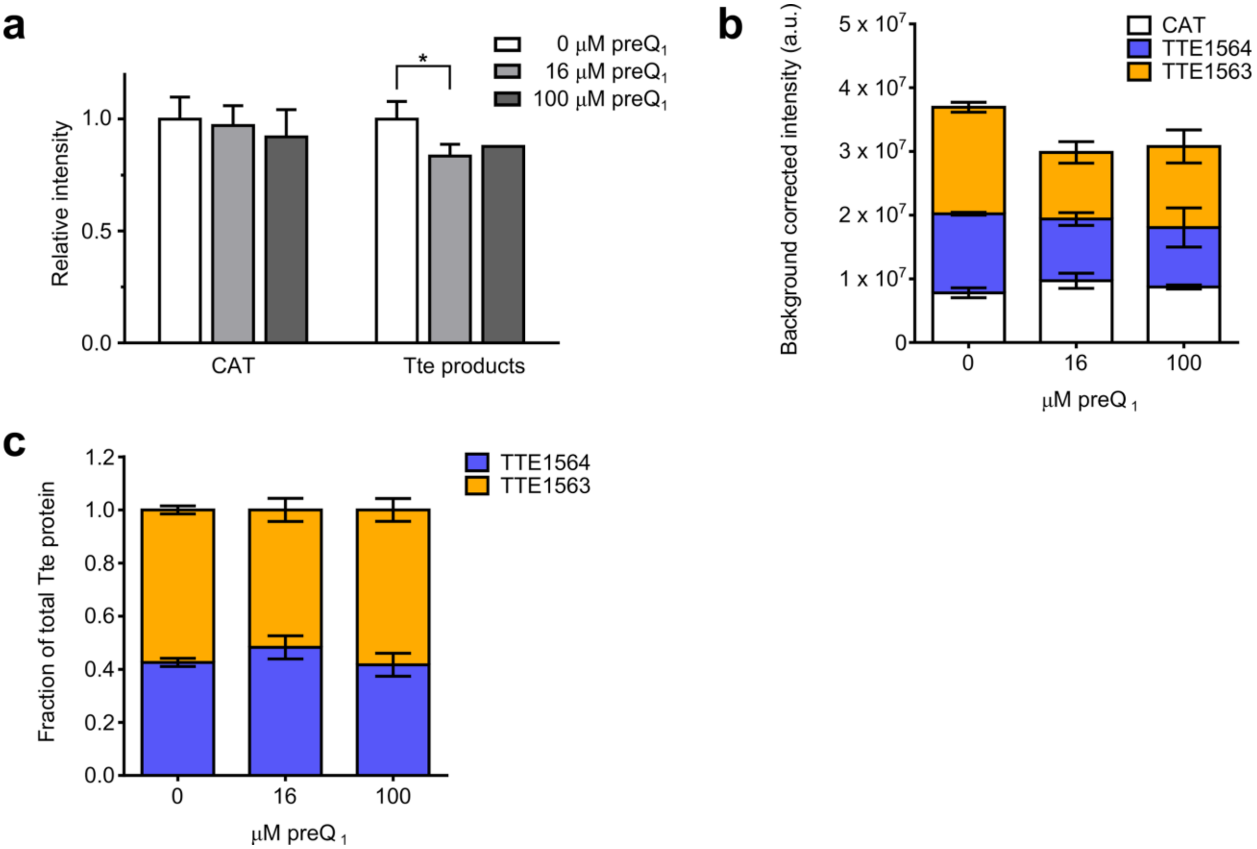

Supplementary Figure 2 | Quantification of the *in vitro* translation of *Tte* mRNA. (**a**) *In vitro* translation reactions containing only CAT or *Tte* mRNA in the presence or absence of saturating concentrations of $preQ_1$. Background corrected band intensities were normalized to the total intensity in the lane and reported relative to the mean of the zero $preQ_1$ reactions (full-length gel is shown in **Supplementary Fig. 13**). The results represent the mean ± s.d. of three replicates, except for the 100 μM $preQ_1$ *Tte* reaction (dark gray bar), which represents a single measurement. (*$P < 0.05$). (**b**) Background-corrected band intensities for the same competition *in vitro* translation experiment shown in **Fig. 1c,d** after normalizing for the cysteine content of each protein (5, 1, 1 for CAT, TTE1564, and TTE1563, respectively). The results represent the mean ± s.d. of a single experiment with three replicates (*$P < 0.05$). See **Supplementary Fig. 11** for full-length gel. (**c**) Relative ratio of TTE1564 and TTE1563 proteins produced as a function of $preQ_1$ concentration from the same experiment shown in **Fig. 1c,d**. The results represent the mean ± s.d. of the ratio of TTE1564 and TTE1563 bands from three replicates. No significant changes in this ratio were observed with increasing concentrations of $preQ_1$, consistent with a tight coupling in expression of the two genes in the operon (full-length gel shown in **Supplementary Fig. 11**).

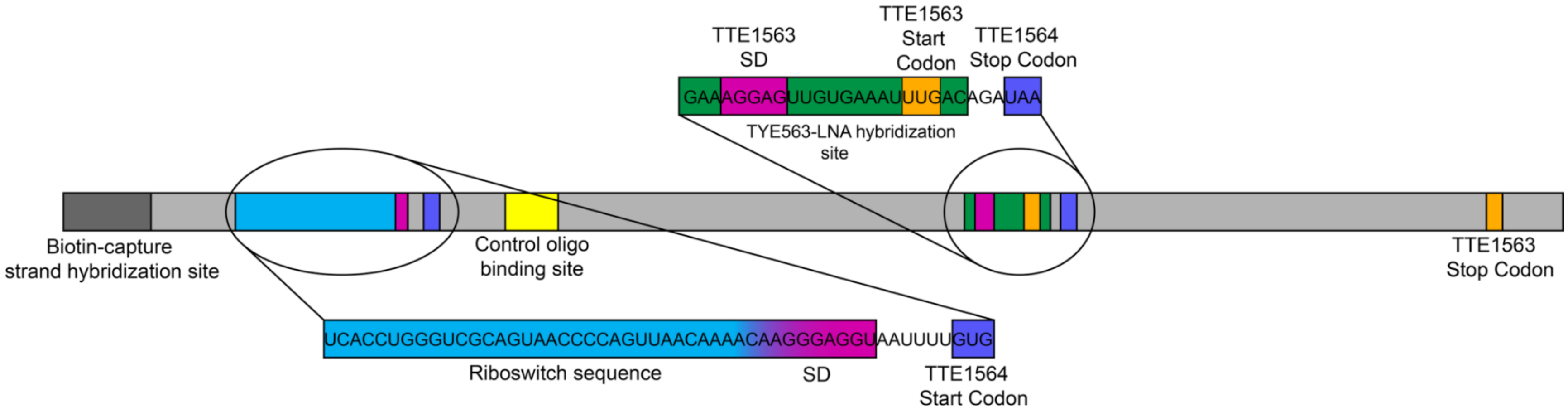

Supplementary Figure 3 | Map of points of interest in the *Tte* mRNA used for SiM-KARTS. mRNA map describing the relative locations of important gene features and hybridization sites used in SiM-KARTS experiments. The cartoon is not drawn to scale.

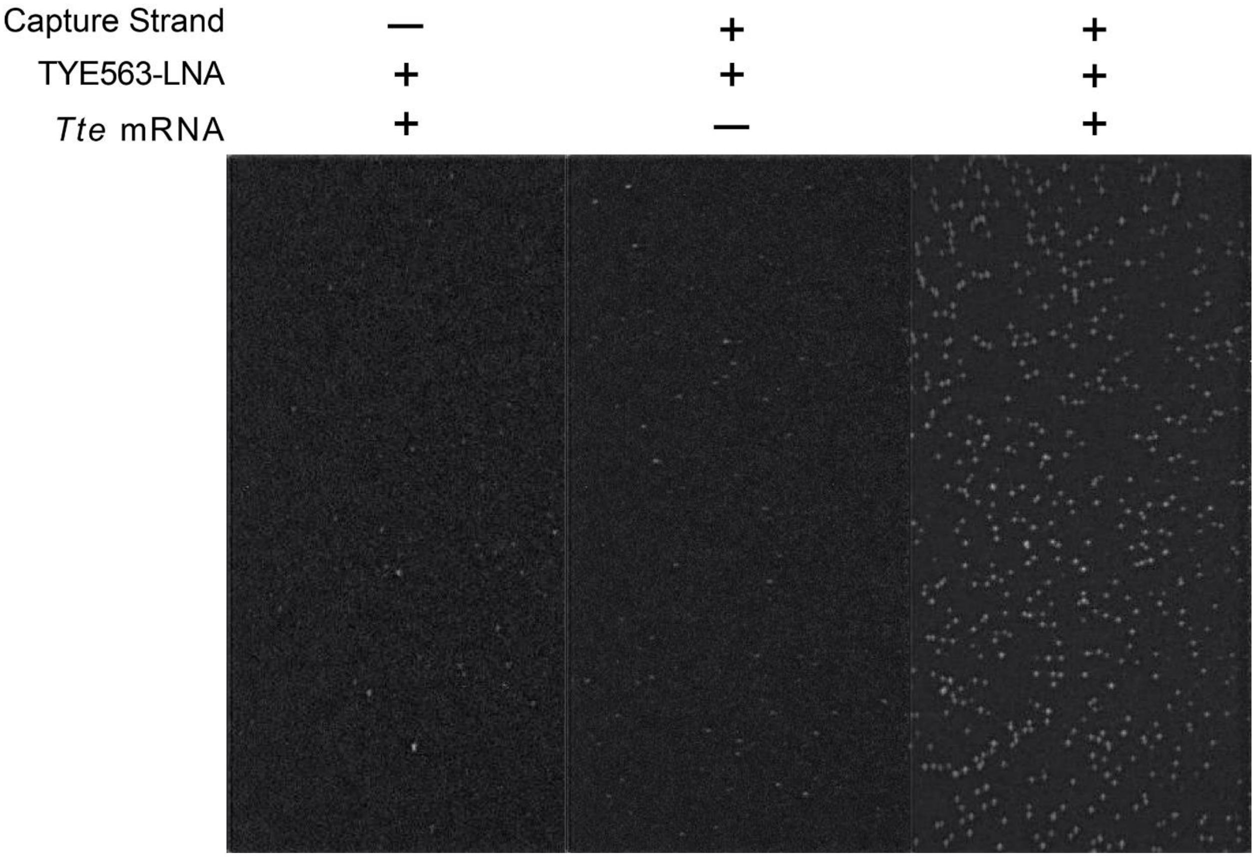

Supplementary Figure 4 | TYE563 emission confirming specific mRNA surface immobilization during SiM-KARTS experiments. Three fields of view displaying the TYE563 emission channel using the prism-TIRFM illumination conditions. *Tte* mRNA, TYE563-LNA and biotin-capture strand were selectively annealed together in SiM-KARTS buffer. Background fluorescence is observed in the left and middle panel, where the biotin-capture strand and mRNA, respectively, were omitted from the complex. TYE563 emission was only observed when all three components were annealed together, shown in the right panel.

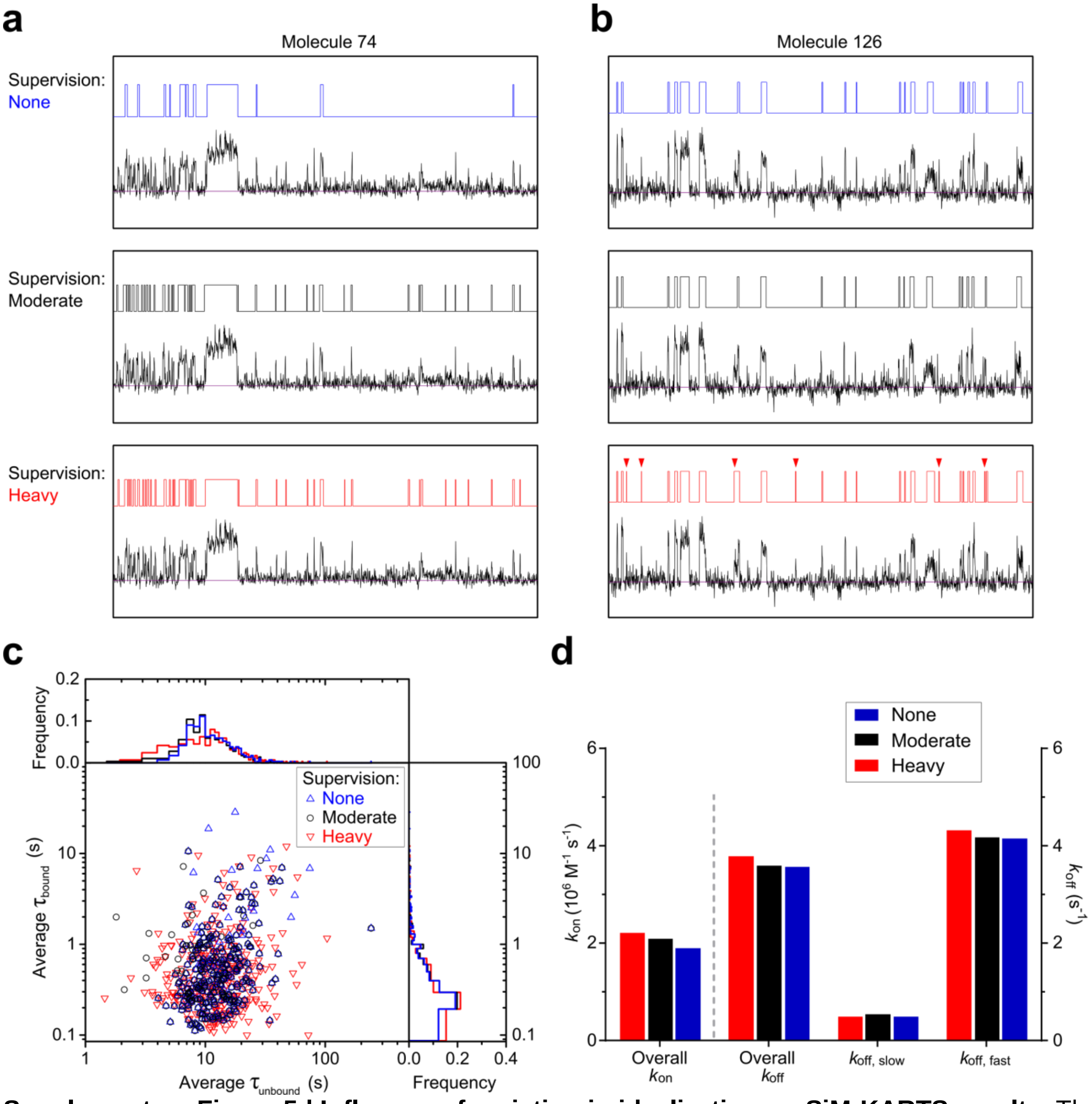

Supplementary Figure 5 | Influence of variation in idealization on SiM-KARTS results. The 288 molecules in the 'No blocking strand' dataset (**Supplementary Fig. 7c**) were idealized to a two-state model using the QuB suite software as described in Methods and **Supplementary Note 2**. (**a**) Example single molecule trace and Hidden Markov idealizations (HMM) from QuB with varying degrees of supervision. The 'Heavy' and 'Moderate' supervision HMMs are identical. (**b**) Example single molecule trace as in **a**. Red arrowheads highlight differences between HMMs generated with 'Heavy' and 'Moderate' supervision. (**c**) Average dwell time correlation plot for the same set of molecules employing different levels of supervision during idealization. Heavy or Moderate adjustment of individual trace idealizations (Heavy, red triangles; Moderate, black circles) increases the range of observed values for average $\tau_{unbound}$ and τ_{bound}, but the center of the distribution is unchanged. (**d**) Comparison of rate constants for the same molecules idealized with different levels of supervision. Idealization-dependent changes in rate constant are modest.

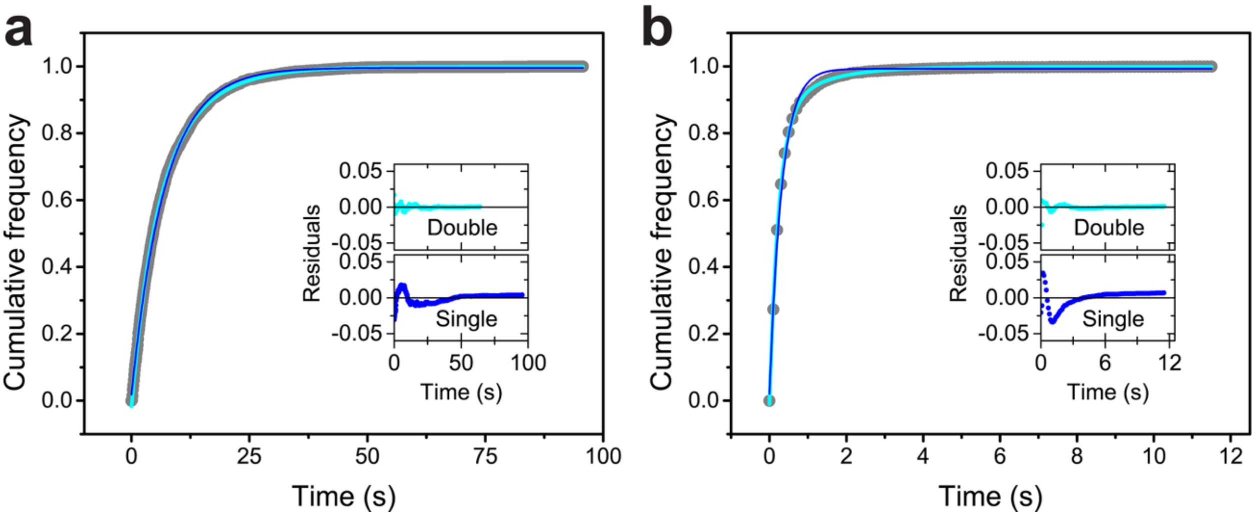

Supplementary Figure 6 | Raw kinetic data of a SiM-KARTS experiment in the absence of ligand. SiM-KARTS plot displaying the cumulative ISIs (**a**) and bound dwell times (**b**) of the anti-SD probe at zero ligand concentration. The dark blue line represents a single-exponential fit, whereas the cyan line represents a double-exponential fit for both plots along with their corresponding residuals.

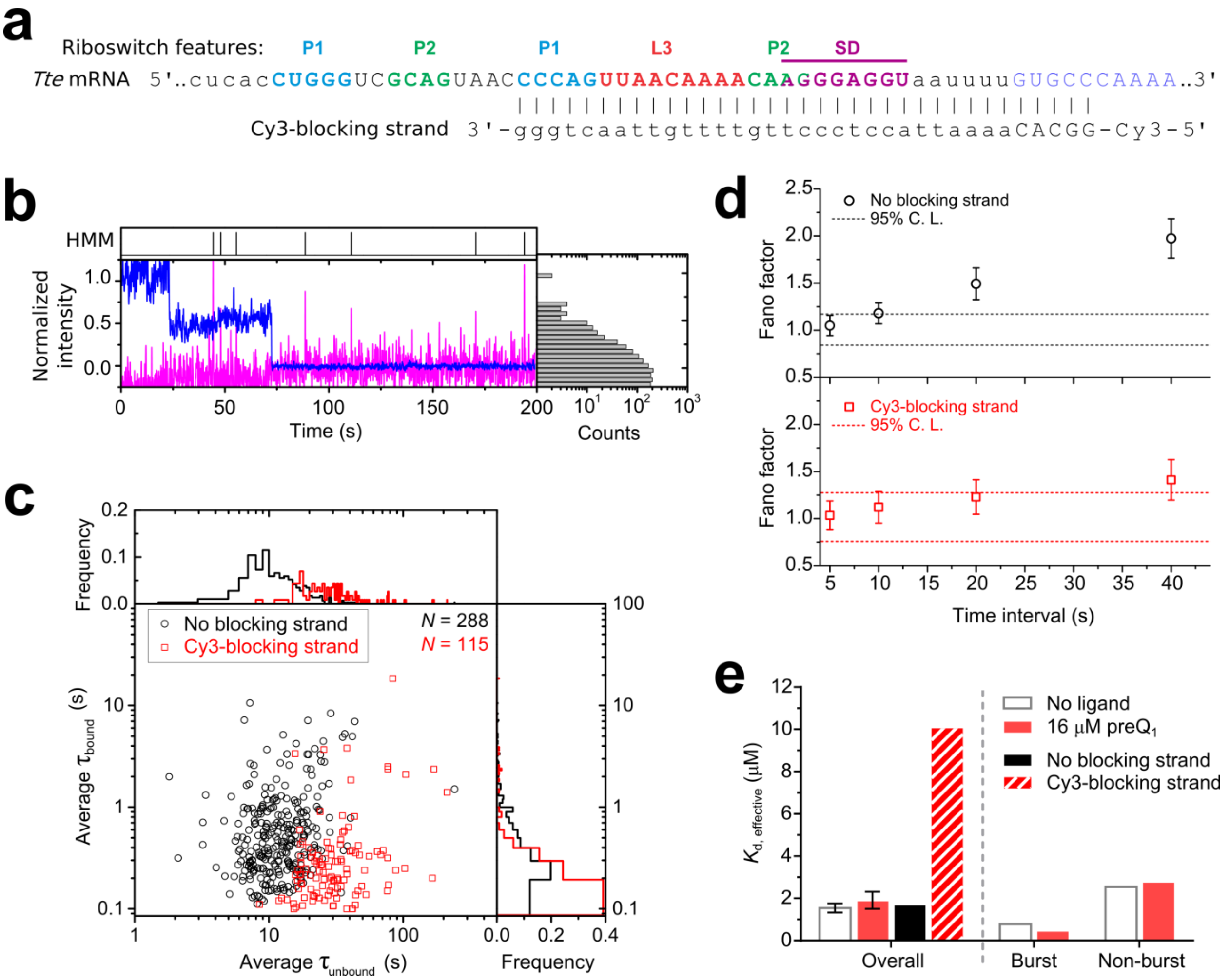

Supplementary Figure 7 | SiM-KARTS experiments on *Tte* mRNA with a blocked expression platform to examine anti-SD probe binding specificity. (**a**) Schematic representation of the binding site of the Cy3-labeled blocking strand. Nucleotides comprising the various structural features of the riboswitch are colored as in **Fig. 1a**. (**b**) Example fluorescence-time trace, Hidden-Markov idealization (HMM), and Cy5 intensity histogram from SiM-KARTS experiments performed with blocking strand. Only molecules that displayed two-step photobleaching in the TYE563/Cy3 channel (blue trace), indicating the presence of both the TYE-563 LNA and the Cy3-blocking strand, were selected for analysis. (**c**) Average dwell time correlation plot for mRNA molecules with and without annealed Cy3-blocking strand. *N* is the number of molecules in the dataset for the respective condition. (**d**) Plot of Fano factors calculated for the anti-SD probe binding data shown in **c.** Dashed lines indicate the expected 95% confidence level for a Fano factor corresponding to a Poisson process, given the number of molecules in the dataset. Error bars indicate the standard deviation of Fano factor values calculated from 100 samplings of the data (see Methods). (**e**) Comparison of the dissociation equilibrium constants ($K_{d,\ effective}$) derived from the measured k_{on} and k_{off} rates (**Supplementary Tables 1** and **2**) for the anti-SD probe and *Tte* mRNA in the absence (No ligand) or presence of saturating (16 µM) $preQ_1$, and for *Tte* mRNA heat annealed in the absence (No blocking strand) or presence of the Cy3-blocking strand. Note that experimental conditions for the “No ligand” and “No blocking strand” were identical, but represent independently collected datasets.

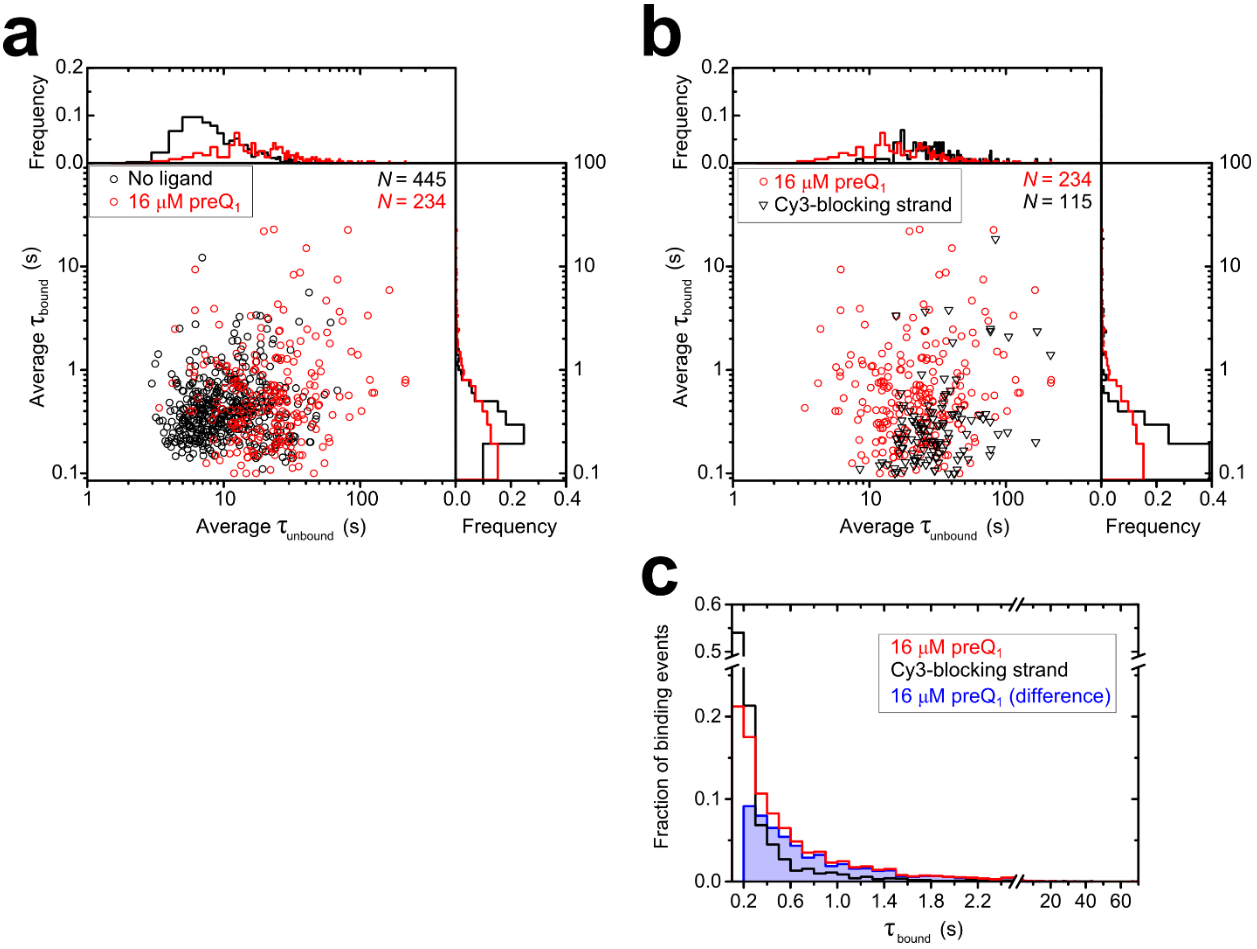

Supplementary Figure 8 | Average bound and unbound times of the anti-SD probe for mRNA molecules in equilibrium SiM-KARTS experiments as a function of ligand or blocking strand. (**a**) The average time each *Tte* mRNA molecule spent with anti-SD probe bound (τ_{bound}) and without anti-SD probe bound ($\tau_{unbound}$) was calculated in the absence of ligand and at 16 µM (saturating) ligand conditions. A slight shift is observed towards longer unbound times in the presence of ligand, but subpopulations within a single ligand condition were not observed. For six molecules in the 16 µM ligand condition, fewer than two binding events were observed in the fluorescence time trace and thus these molecules do not contribute data points to the plot, despite being part of the data set. (**b**) Average τ_{bound} and $\tau_{unbound}$ times for each molecule were calculated for each *Tte* mRNA in the presence of 16 µM preQ1 (reproduced from **a**) and compared to the τ_{bound} and $\tau_{unbound}$ times for *Tte* mRNA whose expression platform was blocked (reproduced from **Supplementary Fig. 7c**). A clear shift is observed towards shorter bound times. (**c**) Histogram of the bound dwell times for binding events observed in the 16 µM dataset presented in **a** (red) versus the blocking strand dataset from **Supplementary Fig. 7c** (black), and the 16 µM $preQ_1$ dataset after removing binding events that last only for a single frame and subtracting a proportional number of binding events according to the bound dwell time distribution observed in the blocking strand dataset, as described in **Supplementary Note 1** (blue). The majority (>60%) of the original binding events remain as specific based on this conservative estimate.

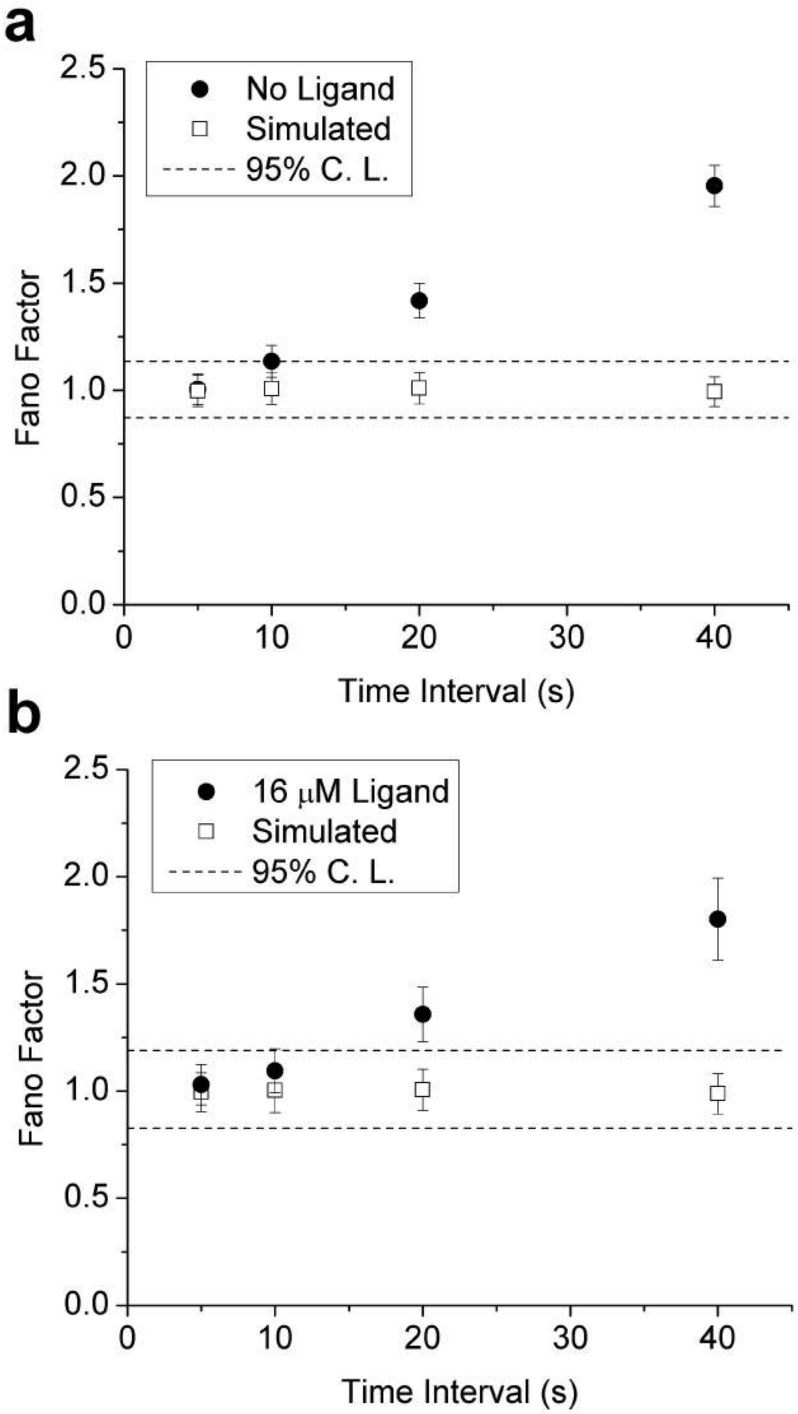

Supplementary Figure 9 | Fano Factor for SiM-KARTS experiments. The Fano factor was calculated across various time intervals for the no ligand (panel **a**) and high (16 μM) $preQ_1$ (panel **b**) datasets. The Fano factor for a simulated dataset with the same overall rate constant as the corresponding experimental condition but derived from a purely Poisson distribution was also calculated and plotted. The dashed lines indicate the 95% confidence level for a Poisson-like process with a specific number of trajectories (N = 445 for no ligand, and N = 234 for high ligand). The SiM-KARTS Fano factor values deviate from 1.0, indicating a non-random underlying distribution, while the simulated Poisson data remain close to 1.0.

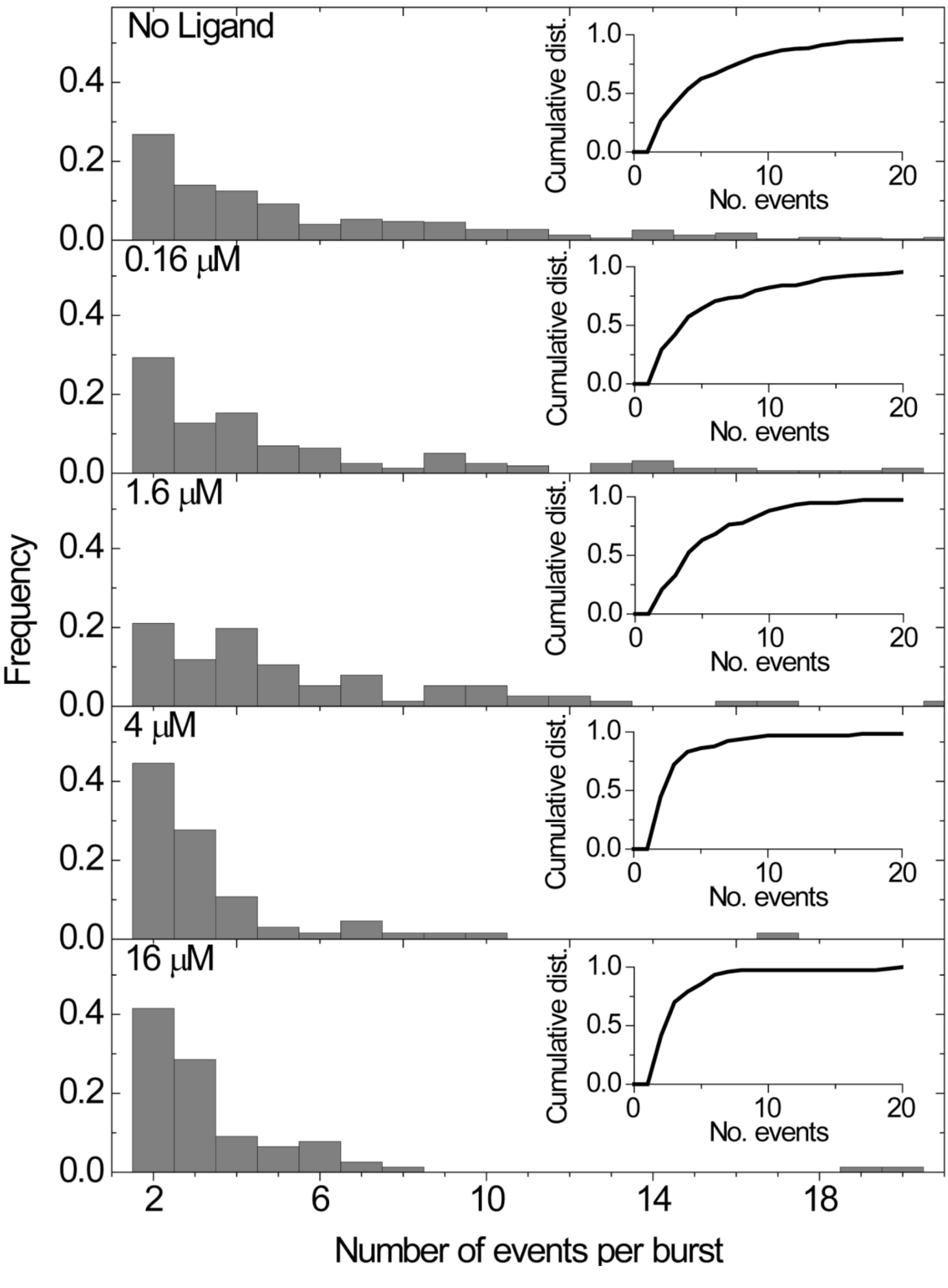

Supplementary Figure 10 | The number of anti-SD probe binding events per burst decreases with increasing ligand concentration. Histograms and cumulative distribution plots (inset) depicting the number of anti-SD probe binding events per burst at different ligand concentrations.

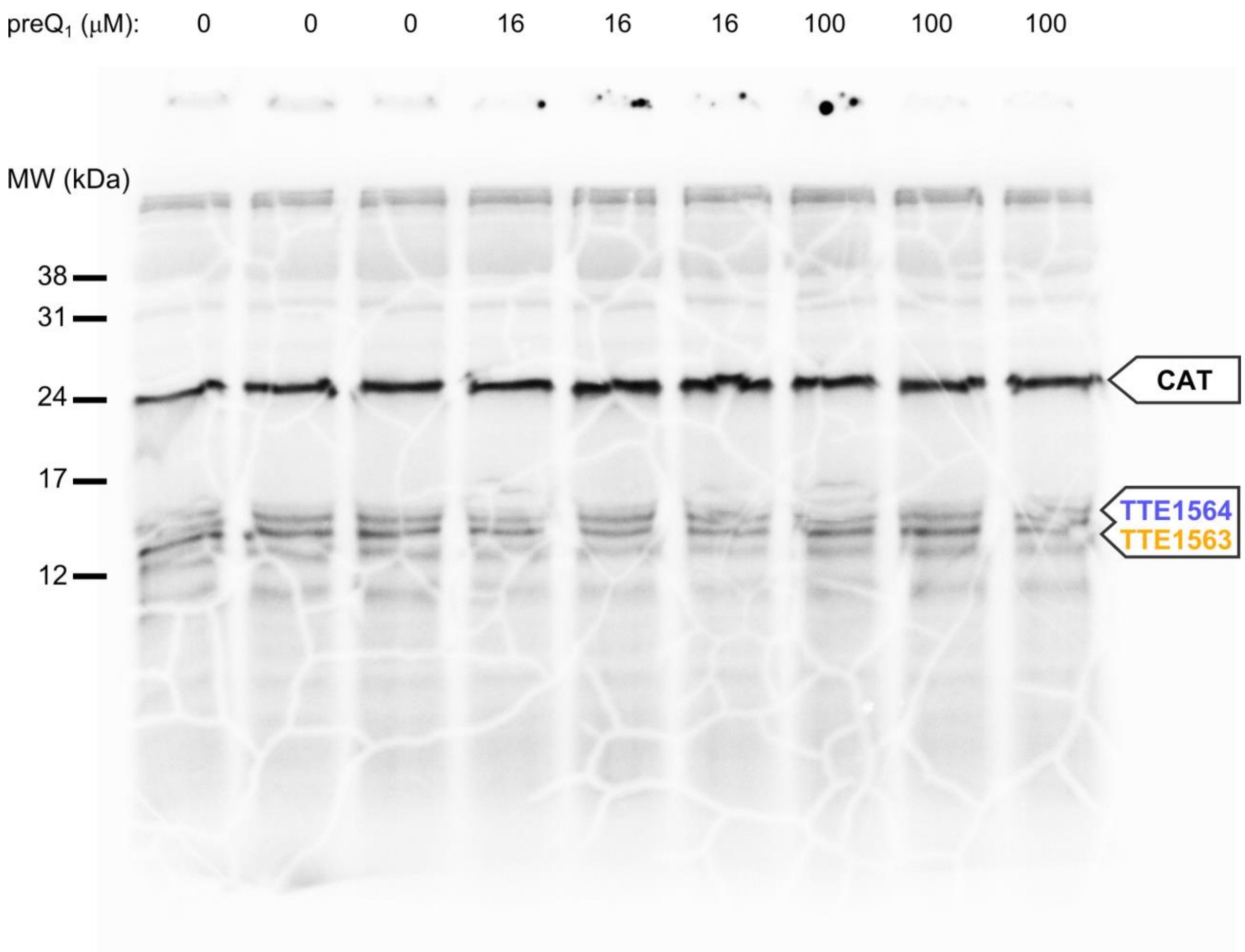

Supplementary Figure 11 | Full gel representing lanes quantified in **Fig. 1d** and **Supplementary Fig. 2b,c**.

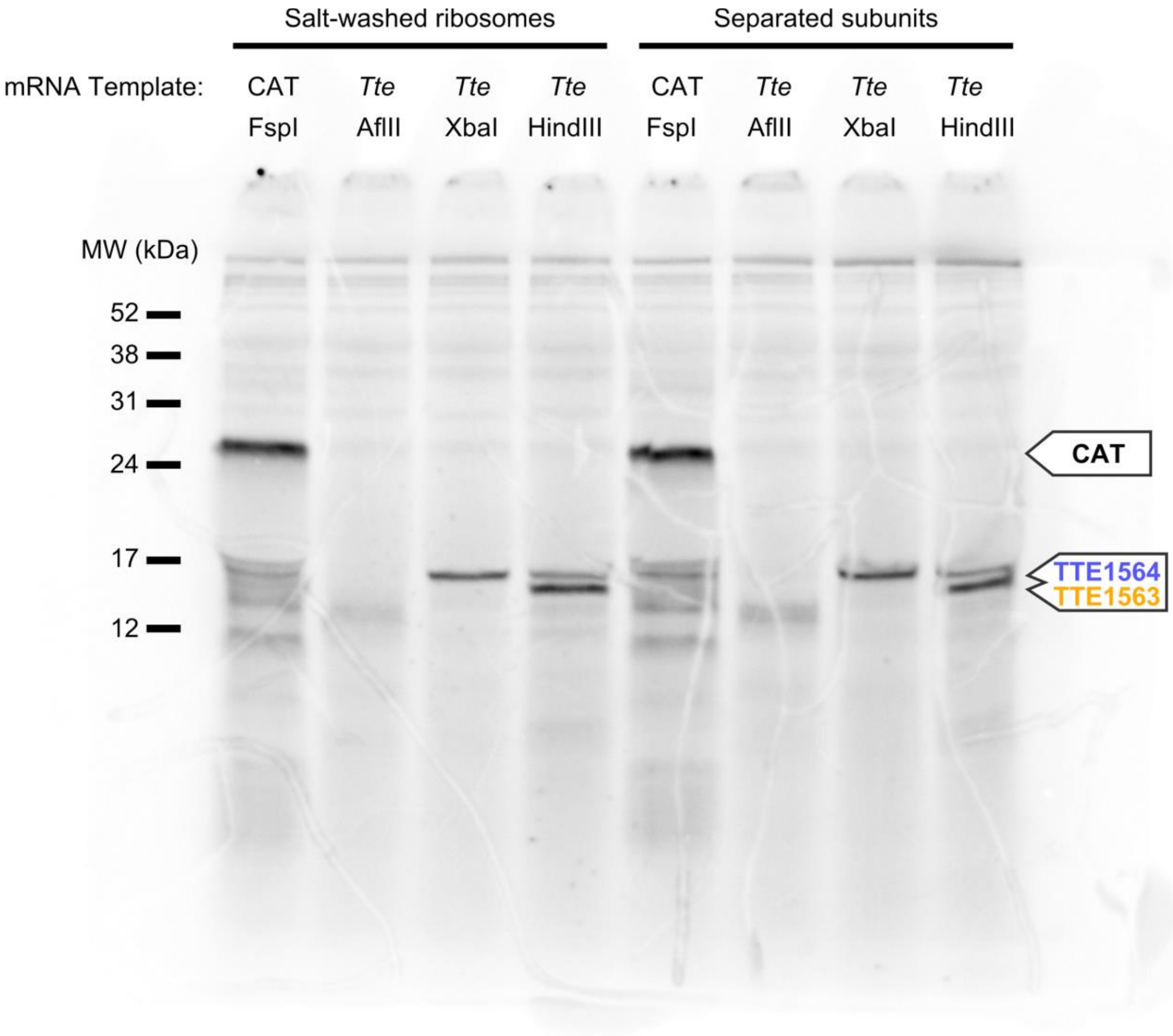

Supplementary Figure 12 | Comparison of the performance of salt-washed ribosomes and separated subunits in *in vitro* translation experiments. Full gel representing lanes quantified in **Supplementary Fig. 1c.**

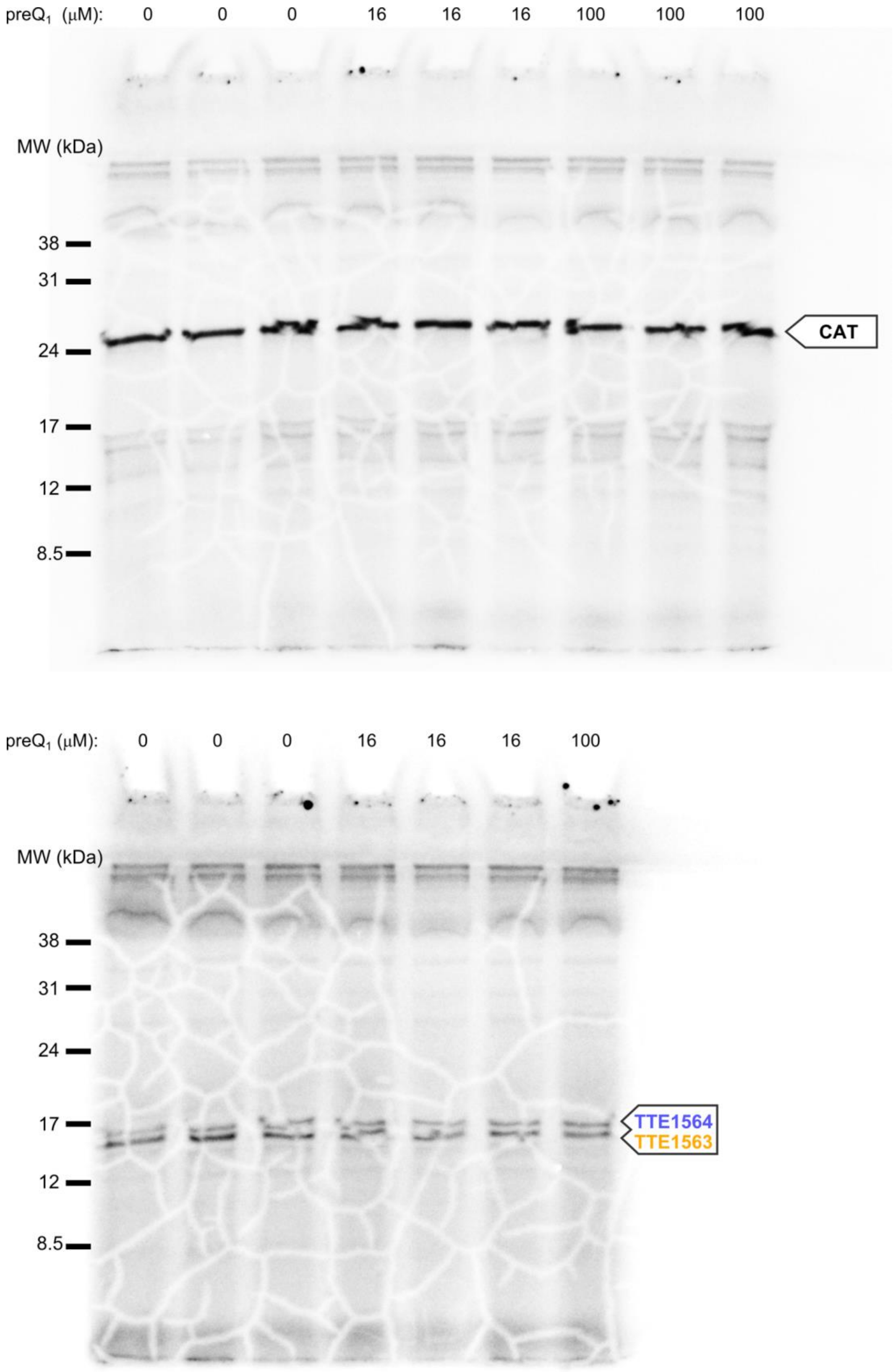

Supplementary Figure 13 | Full gel representing lanes quantified in **Supplementary Fig. 2a**.

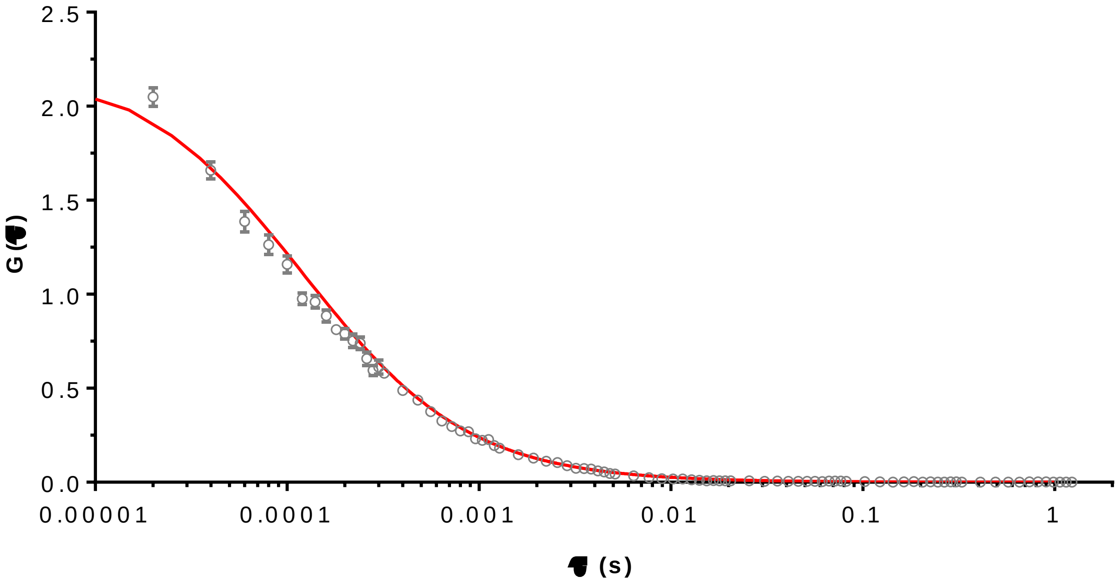

Supplementary Figure 14 | Fluorescence correlation decay plot for the determination of the diffusion coefficient for the anti-SD probe. Coverslips were passivated prior to use with biotinylated BSA and streptavidin as described in the **Methods**. The anti-SD probe was diluted to 2.5 nM in SiM-KARTS buffer, a small aliquot was placed on the washed and dried coverslip, and measurements were performed at room temperature on an Olympus IX81 inverted microscope with an ISS ALBA 5 confocal system (Champaign, IL). Ten replicate traces of 30 seconds each were acquired and sampled at 50 kHz. Ten replicate traces of 30 seconds each were acquired, and sampled at 50 kHz. The data from each replicate were averaged and fit in PyCorrFit v0.9.1 (http://pycorrfit.craban.de/)[1] with a correlation function for three-dimensional free diffusion with a Gaussian laser excitation profile (elliptical), including a triplet component as described in the **Methods**. Data points and error bars (gray) represent the mean of ten replicate measurements ± the standard error of the mean. The fit of the average correlation curve is plotted in red.

Condition	Median burst duration (s)		k_{on} (10^6 M^{-1} s^{-1})	ISI $t_{1/2}$ (s)	k_{off} (s^{-1})[c]
No Ligand	7.95	Burst [a]	4.87 ± 0.02	2.85 ± 0.01	3.82 ± 0.07
		Non-burst [a]	1.47 ± 0.01	9.45 ± 0.03	3.74 ± 0.05
		Overall [b]	*2.4 ± 0.3*		*3.7 ± 0.2* (76 ± 5%)
0.16 μM	7.5	Burst [a]	5.23 ± 0.03	2.65 ± 0.01	2.83 ± 0.02
		Non-burst [a]	0.974 ± 0.002	14.2 ± 0.1	2.40 ± 0.02
		Overall [b]	*1.74 ± 0.4*		*2.8 ± 0.4* (79 ± 4%)
1.6 μM	8.3	Burst [a]	5.18 ± 0.04	2.67 ± 0.02	2.22 ± 0.04
		Non-burst [a]	1.22 ± 0.01	11.4 ± 0.1	2.43 ± 0.03
		Overall [b]	*2.3 ± 0.5*		*2.3 ± 0.2* (70 ± 7%)
4 μM	3.1	Burst [a]	4.65 ± 0.02	2.98 ± 0.01	2.45 ± 0.04
		Non-burst [a]	0.985 ± 0.002	14.1 ± 0.1	2.84 ± 0.03
		Overall [b]	*1.1 ± 0.2*		*2.2 ± 0.2* (76 ± 1%)
16 μM	2.8	Burst [a]	6.73 ± 0.05	2.06 ± 0.02	2.9 ± 0.1
		Non-burst [a]	0.888 ± 0.002	15.6 ± 0.1	2.46 ± 0.06
		Overall [b]	*1.1 ± 0.1*		*2.1 ± 0.4* (80 ± 6%)

a. Values were calculated from single exponential fits of the pooled data from all experiments for a given condition. The reported error is the standard error of the fit.

b. Values represent the average ± the standard error of the mean of three independent experiments.

c. Values represent the weighted average of the fast and slow rate constants derived from a double exponential fit. Percentages in parentheses indicate the contribution of the fast dissociation rate constant to the overall k_{off}.

Supplementary Table 1 | Kinetic parameters extracted from SiM-KARTS and burst analysis

The cumulative distribution plots corresponding to the ISI times inside and outside of the bursts shown in **Fig. 4b** were fit with an exponential function from which the ISI half-lives ($t_{1/2}$) were calculated. No significant change is observed in $t_{1/2}$ values of the ISIs inside the burst; by contrast, a notable increase is observed in the ISIs outside the bursts as ligand concentration is increased. Similarly, the burst duration decreases with increasing ligand concentration.

Condition		k_{on} (10^6 M^{-1} s^{-1})	k_{off} (s^{-1})	$K_{d,\ effective}$ (μM)
No blocking strand	*Overall*	*2.1*	*3.6 (84%)*[a]	*1.7*
	Fast	--	4.2	
	Slow	--	0.54	
Heat anneal with Cy3-blocking strand	*Overall*	*0.82*	*8.3 (84%)*[a]	*10*
	Fast	--	9.6	
	Slow	--	1.1	

a. Values represent the weighted average of the fast and slow rate constants derived from a double-exponential fit. Percentages in parentheses indicate the contribution of the fast dissociation rate constant to the overall k_{off}.

Supplementary Table 2 | Kinetic parameters in the presence and absence of a blocking strand. The cumulative frequency distributions of all unbound and bound dwell times for SiM-KARTS experiments shown in **Supplementary Fig. 7** were fit with single (k_{on}) or double (k_{off}) exponential association functions. In the presence of a Cy3-blocking strand that occludes the riboswitch expression platform (containing the TTE1564 SD, **Supplementary Fig. 7a**), anti-SD probe binding events are infrequent and short-lived. The $K_{d,\ effective}$ is derived using the measured rate constants (k_{off}/k_{on}) for probe binding. When the expression platform is occluded, the affinity of the anti-SD probe is solely due to any remaining binding sites elsewhere in the mRNA, and is greatly reduced.

Supplementary Note 1

Choice of Cy5 probe sequence for SiM-KARTS experiments on the preQ$_1$ riboswitch in its native context

The SiM-KARTS technique exploits the transient and repeated binding of a short, fluorescently labeled probe oligonucleotide to interrogate the structure of a conformationally dynamic site of interest in an RNA of arbitrary size to report on structural changes at that site through changes in the probe's binding and dissociation kinetics.

While we could have chosen to use a probe of different length with perfect complementarity, we chose instead to use the anti-Shine Dalgarno sequence at the 3' end of the 16S rRNA that *Thermoanaerobacter tengcongensis* uses to bind the Shine-Dalgarno sequences of bacterial mRNAs to initiate translation. By choosing this sequence, we effectively created a highly simplified *in vitro* mimic of the bacterial ribosome. The use of the 16S rRNA sequence, which is highly, though not exactly, complementary to the riboswitch expression platform, allows us to recapitulate the interaction between the ribosome and the mRNA. This alleviates the concern that if we were to use a different probe sequence with perfect complementary and possibly slightly elevated site-specificity, we might inadvertently alter the nature of what is likely a carefully balanced interplay between the SD and anti-SD sequences, which evolved together to bring about the regulatory control needed by the bacterium.

Anti-SD probe binding frequency and duration are greatly decreased in the presence of a blocking strand

We are not able to entirely rule out the possibility that some of the anti-SD probe binding events we observed are due to binding at sites other than the TTE1564 Shine-Dalgarno (SD), the expression platform of the riboswitch. In fact, the presence of fluorescence traces exhibiting multistep TYE563 photobleaching indicates that it is possible for the TYE563-LNA to improperly hybridize at a site other than the TTE1563 SD and start codon, thus allowing binding by the anti-SD probe at the downstream TTE1563 SD for that subset of *Tte* mRNA molecules. However, the low number (5-15%) of fluorescence traces exhibiting such multistep TYE563 photobleaching, even in the presence of stoichiometric excess of TYE563-LNA, strongly suggests that, although off-target sites for the TYE563-LNA exist, misannealing of the TYE563-LNA is rare, and thus anti-SD probe binding at the TTE1563 SD is likely negligible.

Because the Cy5-labeled anti-SD probe has the same sequence as the 3' end of the 16S rRNA, it has the potential to bind transiently to true SD sequences, as well as SD-like sequences in the mRNA. Indeed, recent work by Li and Weissman[2] has indicated a biologically important role for binding of the 16S rRNA to SD-like sequences in the open reading frame during translation. To assess the potential for the Cy5-labeled anti-SD probe to bind at other sites in the *Tte* mRNA, we performed equilibrium SiM-KARTS experiments as described in the main text with an additional Cy3-labeled blocking strand present during initial complex heat annealing and dilution before immobilization on the slide surface. The Cy3-blocking strand hybridizes to the expression platform and a large portion of the riboswitch aptamer domain, effectively preventing binding by the anti-SD probe at the TTE1564 SD (**Supplementary Fig. 7a**).

The fluorophores TYE563 and Cy3 attached to the LNA and blocking strand, respectively, have similar fluorescence emission profiles and thus traces that, in this experiment, exhibited two-step photobleaching indicate the presence of both the Cy3-blocking strand and the TYE563-LNA (**Supplementary Fig. 7b**). For these mRNAs, both the TTE1564 SD and TTE1563 SD are sequestered and so any observed binding events are due to binding at other sites on the mRNA. In SiM-KARTS experiments where the Cy3-blocking strand is present, the frequency of anti-SD probe binding events dramatically decreases for the majority of molecules, and the anti-SD probe stays bound for shorter periods; this is reflected in a marked shift towards longer average unbound dwell times, and a decrease in the average bound dwell time

(**Supplementary Fig. 7c**). Importantly, analysis of the Fano factor for different time intervals reveals that, unlike the 16 µM data (**Supplementary Fig. 9b**), anti-SD probe binding to *Tte* mRNA heat-annealed in the presence of Cy3-blocking strand is a Poisson process and does not occur in bursts (or, at minimum, not on a comparable timescale).

Analysis of the binding and dissociation rate constants (k_{on} and k_{off}) shows that the on-rate is decreased in the presence of the Cy3-blocking strand, and the off-rate is significantly increased, approaching the limit of the time resolution used in our experiments (**Supplementary Table 2**). Taken together, these data indicate that under the conditions of the SiM-KARTS experiments presented in the main text, binding of the anti-SD probe at sites in the mRNA other than TTE1564 SD occurs infrequently, and that the probe is weakly bound and dissociates quickly. In contrast, the k_{off} values measured in the in the presence of saturating (16 µM) preQ_1 and absence of the Cy3-blocking strand are several fold slower than would be expected if all observed binding events were due to probe binding at sites other than the TTE1564 SD (**Supplementary Tables 1, 2**) and is similarly evidenced by a population shift towards shorter average τ_{bound} times (**Supplementary Fig. 8b**). Both of these observations lend support to the assertion that the anti-SD probe primarily reports on accessibility of TTE1564 SD and that binding at other sites would contribute to only a slight underestimation of k_{on} and overestimation of k_{off}. This finding is perhaps captured most clearly by the change in $K_{d,\ effective}$ for *Tte* mRNA heat-annealed in the presence of Cy3-blocking strand (**Supplementary Fig. 7e, Supplementary Table 2**), where the affinity of the anti-SD probe for the *Tte* mRNA decreases significantly when the expression platform is blocked, to a level lower than what is seen in the presence of saturating ligand, in both Burst and Non-burst periods.

This observation supports the assertion that the majority of binding events observed, even under saturating ligand conditions, are indeed genuine and SD specific. For example, one can assume that the bound dwell time distribution observed in the presence of 16 µM ligand is in fact the sum of "genuine" binding events and "non-specific" binding events (**Supplementary Fig. 8c**, red histogram). One may also – conservatively – assume that the bound dwell time distribution for binding events observed for *Tte* mRNA annealed with the blocking strand represents the expected contribution of probe binding at sites other than the riboswitch expression platform i.e., "non-specific" binding (**Supplementary Fig. 8c**, black histogram). Because relative distribution of bound dwell times for the blocked dataset is skewed towards binding events lasting only a single frame, if this dwell time distribution is scaled such that the number of single-frame binding events is equal to that observed in the 16 µM dataset, then subtracting the scaled, blocked dataset distribution from the 16 µM dataset distribution should remove all "non-specific" binding events (and possibly also a good fraction of brief specific binding events; **Supplementary Fig. 8c**, blue histogram). After this correction for possible background, a majority (>60%) of binding events in the original distribution still remain. We note that this is in fact overly-conservative because it assumes that all binding events lasting only one frame in the 16 µM dataset are non-specific, which is unlikely to be the case, and thus overcorrects for the amount of non-specific binding.

Supplementary Note 2

Minimal trace data preprocessing and idealization in QuB

Prior to idealization using a two-state Hidden Markov Model (HMM), single molecule fluorescence time traces were preprocessed using a custom Matlab script to provide a rough normalization of the Cy5 fluorescence intensity across all molecules in the dataset. This is necessary because an overly large range of intensities that represent the bound state for different molecules will make it difficult to assign characteristic intensity values for the bound and unbound states in HMM analysis. In the present study, a wide range of Cy5 fluorescence

intensities were observed for the probe in the bound state. Modest variability in the Cy5 intensities observed for molecules in the same experiment results from uneven laser illumination and excitation across the field of view during acquisition, and variability across experiments is due to the inherently arbitrary units of a fluorescence intensity. During preprocessing, the mean (m) and standard deviation (σ) of the Cy5 fluorescence intensity was determined across all frames in a given trace. Then, the Cy5 intensity for each frame was divided by the quantity $m + n\sigma$, where the multiplying factor n is determined empirically for each experimental system. For the experiments presented in the current study, $n = 4$. In principle, other more sophisticated methods of normalization that result in comparable intensity values for the bound and unbound state between different molecules can be used, provided that assumptions inherent in HMM analysis are not violated (e.g., a Gaussian noise profile)[3]. Additionally, in cases where data were pooled from experiments in which the observation time (i.e., movie length) was significantly different, the traces were truncated so that all molecules in the dataset were analyzed over the same observation time. Because of the behavioral heterogeneity common to single molecule studies, it is important to ensure in this way that molecules contribute equally to subsequent analyses.

After preprocessing, normalized trace data were compiled into a single, segmented file and loaded into the QuB suite (v2.0.0.22, University at Buffalo). The camera integration time (100 ms in this study) was used for the sampling time. Each trace was treated as an individual segment. A two-state model was constructed with approximate estimates of the forward (unbound → bound) and reverse (bound → unbound) rates; 0.1 and 2.5, respectively, were used as starting rate estimates in the current study. After constructing the model, the mean amplitude and standard deviation of each state were estimated over all segments in the file using the Amps function. Because the signal-to-noise ratio varies between different molecules, the standard deviation for the unbound state was fixed at a relatively high value (between 0.22 and 0.3 in the present study) to avoid initial over-fitting of the bound state. All of the segments in the file were then simultaneously idealized using a fixed standard deviation for the unbound state by the segmental k-means (SKM) algorithm.

Slight variability in trace idealization is well tolerated in SiM-KARTS

Analyzing all of the segments in this way produces a reasonable idealization for the majority of molecules; however, additional adjustment of individual traces is frequently necessary. We refer to the degree to which the idealization for each molecule is scrutinized as supervision. To examine the effects of variations in idealization, the “No blocking strand” dataset (**Supplementary Note 1, Supplementary Fig. 7c**) was independently idealized three times, exercising a different level of supervision each time (None, Moderate, or Heavy). The initial idealization of traces, as described above without any further adjustment, is referred to as unsupervised idealization. As seen in the example trace in **Supplementary Fig. 5a,** the initial unsupervised idealization (None, blue HMM) sometimes inadequately fits genuine binding events. In these instances, the idealization for the specific molecule is repeated after adjusting the model’s starting parameter estimates (Moderate or Heavy supervision). We found that re-estimating the mean amplitudes and standard deviations of each state for the specific trace using the Amps function, or simply decreasing (or, in the case of over-fitting, increasing) the standard deviation of the unbound state, and then repeating the idealization is sufficient to achieve an accurate HMM fitting (**Supplementary Fig. 5a**, Moderate black and red HMMs).

When exercising Moderate supervision, the idealization for a given molecule was adjusted only when there were obvious deficiencies in the HMM fitting as found in the example in **Supplementary Fig. 5a**. Similarly, the HMM for a given molecule was often unchanged from the initial unsupervised idealization (**Supplementary Fig. 5b**; compare None, blue, and Moderate, black, HMM fits). By contrast, when exercising Heavy supervision, very close attention was paid to each trace’s idealization, which led to frequent adjustment in an effort to

capture every increase in fluorescence intensity that could reasonably be interpreted as a binding event (**Supplementary Fig. 5b**; red arrowheads highlight the differences between idealizations in which Heavy, red, or Moderate, black, supervision was employed). This extent of refinement of a given trace's idealization occasionally required changing additional parameters such as the maximum number of iterations used in the fitting algorithm, adjusting the initial rate estimates used by the model, or examining the histogram of fluorescence intensities to visually estimate the center of the bound state amplitude (mean intensity) and possibly employing a fixed large value for the standard deviation for the bound state intensity. The latter was often required if a trace exhibited a relatively small number of short-lived binding events with dramatically different intensities, leading to a very broad distribution of bound state intensities that was too sparsely populated within the observed time window to be fit well by the algorithm.

From the plot of average dwell times, one can clearly see that Moderate and Heavy supervision of the idealization process results in slightly greater dispersion of average unbound dwell times and a slight compaction of average bound state dwell times, as would be expected after correction of under- or over-fitting of binding events, and under-fitting of short bound dwell times (**Supplementary Fig. 5c**). However, the overall distributions remain largely unchanged. This fact is perhaps best reflected by the calculated rate constants: while there are slight differences in the calculated rate constants depending on the degree of supervision used, overall the calculated rates are little affected (**Supplementary Fig. 5d**).

With the obvious exception of **Supplementary Fig. 5**, all of the SiM-KARTS data in the current study were idealized employing what we describe above as Moderate supervision. Completely unsupervised idealization is clearly insufficient in the case of some molecules, as demonstrated above (**Supplementary Fig. 5a**). While it is expected that some genuine binding events will be missed with only Moderate supervision, the use of Heavy supervision during idealization introduces an undesirable degree of subjectivity into the analysis, and thus will likely be more prone to bias and over-fitting. Given that the level of supervision ultimately had little impact on the final rate constant analysis (**Supplementary Fig. 5d**), we conclude that the analysis of SiM-KARTS data is robust, provided there is a sufficient number of molecules in each dataset, and that slight variability in idealization (e.g., the occasional missed binding event) is well tolerated.

Supplementary Note 3
Considerations for rate constant determinations

Key to simplifying the interpretation of binding event data is establishing experimental conditions under which measurements of probe dissociation is not limited by photobleaching and probe binding is not limited by diffusion. Given the relatively fast rates of dissociation and the concentration of anti-SD probe (dynamically refreshed from the bulk solution), Cy5 fluorophore bleaching does not affect the observed off rates[4,5]. Following a similar method to that described by Dupuis *et al.*[6], we measured the diffusion coefficient of the anti-SD probe (D_{probe}) in SiM-KARTS buffer to be 1.6 ± 0.2 × 10^{-6} $cm^2\,s^{-1}$ using fluorescence correlation spectroscopy. The diffusion limited rate-constant k_{diff} in terms of $M^{-1}\,s^{-1}$ can be estimated using Eq. 1:

$$k_{\text{diff}} = 4\,\pi r_{\text{RNA}} D_{\text{probe}} \frac{N_{\text{A}}}{1000\ \text{cm}^3} \qquad \textbf{(1)}$$

where N_{A} is Avogadro's number, and the radius of the 12 nt anti-SD probe, r_{RNA}, is taken to be approximately 24 Å, a value that is slightly larger than expected for a duplex of the same general length (20.5 Å for an 8-mer or 12-mer helix[7])[6]. This predicts a diffusion-limited rate ≈ 3 × 10^9 $M^{-1}s^{-1}$, which is significantly faster than the values we measured for k_{on} (that are on the order

of 10^6 M^{-1} s^{-1}, see **Fig. 2d, Supplementary Table 1**), demonstrating that the hybridization of the anti-SD probe is not limited by diffusion.

Another important aspect of our SiM-KARTS approach is that its true power is derived from the relative changes in binding kinetics of the probe, which is used to report on the conformational state of a larger RNA. Although we do not measure the concentration dependence required to rigorously determine the bimolecular rate constant k_{on} according to the linear relationship:

$$\frac{1}{\tau_{\text{unbound}}} = k_{\text{on, observed}} = k_{\text{on}}c \qquad \textbf{(2)}$$

where *c* is the concentration of anti-SD probe, there is a strong precedent in the literature demonstrating this relationship for related experimental systems involving short nucleic acid duplexes[5,6]; because our k_{on} values (presented in **Fig. 2d** and **Supplementary Table 1**), determined from experiments at a single concentration of anti-SD probe, are in excellent agreement with k_{on} rate constants determined previously under similar ionic conditions, (e.g., Jungmann *et al.*[5] 2.3×10^6 $M^{-1}s^{-1}$ for a 9 bp duplex with 600 mM NaCl), we conclude that our values are a good approximation of the true bimolecular k_{on} in our system. Furthermore, the main effect of increasing ligand is a clear change in the burst behavior of the *Tte* mRNA, and is reflective of the intrinsic, unimolecular folding of the *Tte* mRNA; identification and analysis of these relative trends do not rely on, and are not sensitive to, the absolute values of the rate constants.

Ligand-dependent changes in anti-SD probe binding kinetics are consistent with expectations based on previous studies of short duplex annealing kinetics

Integral to the regulation exerted by the riboswitch is the inherent nuance in differences of hybridization kinetics between a SD•anti-SD duplex with 6 versus 8 possible base pairing interactions (**Supplementary Fig. 1a**). As such, it is useful to discuss recent work examining the kinetics of such short complementary oligonucleotides.

In a study by Dupuis *et al.*, hybridization kinetics were observed by smFRET for a series of short, fully complementary duplex DNAs, revealing that from a 6-bp to an 8-bp duplex the τ_{bound} increased by approximately 100-fold[6]. In addition, the authors observed a linear relationship between increasing duplex length and decreasing $\Delta G°$ of hybridization, where $\Delta G°$ decreased by ~1 kcal mol^{-1} bp^{-1} as one might intuitively expect – formation of a longer duplex is more favorable than formation of a shorter one. Their examination of perfectly complementary duplexes thus demonstrates that a difference of just two basepairs has an outsized impact on duplexes of this length. Applying this observation to our own study, it is reasonable to expect ΔG to change by +2 kcal mol^{-1} as two basepairs in the SD sequence become unavailable due to ligand binding. Interestingly, the prior study found that the effect of changing duplex length was primarily on k_{off} (140 s^{-1} versus 0.40 s^{-1} at relatively low ionic strength for perfect 6 and 8 bp DNA duplexes, respectively) and showed that k_{on} only slightly decreased with increasing duplex length (from 5.0×10^6 $M^{-1}s^{-1}$ to 3.5×10^6 $M^{-1}s^{-1}$). However, such specifics are undoubtedly context dependent since it has been known since the 1970's that successful binding events are initiated by a few metastable basepairing interactions, followed by zippering of the remaining basepairs (as summarized in the recent Ref. [8]), and they are influenced by probe and target secondary structures (see, e.g., Ref. [9]).

We can draw closer parallels to the single molecule FRET studies of Cisse *et al.*[10] examining the position-dependent effects of internal mismatches on the hybridization and dissociation kinetics of a 9-bp duplex. In a 9-bp DNA duplex with similar pattern of weak and strong basepair interactions as our anti-SD probe, internal mismatches resulting in less than 7 contiguous basepairs showed a 30-fold increased k_{off} and 100-fold decreased k_{on}. The authors

also found the same to be true for duplex RNA. Their findings highlight the sensitivity of the binding and dissociation kinetics to changes in the number of basepair interactions in this length regime, leading them to postulate that the observed 7-bp complementarity may play a role in target discrimination by the seed sequence of microRNA.

In summary, our measured rate constants are well within the expected range observed in previous studies[5,6,10 and ref. therein], and it is very reasonable to expect that there will be robust and measurable changes in the hybridization and dissociation kinetics for duplexes that when annealed are 6 or 8 bp, respectively. The exact direction and magnitude of a change, however, will be difficult to predict. Our system is more complex than earlier studies examining short duplex binding kinetics. Of course, formation of helix P2 will dynamically and competitively exclude part of the SD sequence our anti-SD probe binds, and there is the possibility of interactions between the probe and adjacent secondary and tertiary structure in the mRNA, which may further modulate the on- and off-rates (for example, we discuss the possibility for co-axial stacking of P2 with the anti-SD probe-SD helix leading to a slower than expected k_{off}, see main text). Additionally, other factors such the sequence dependence on the opening and closing rates of the helix's closing basepair[11], and the sometimes non-intuitive stabilization afforded by different combinations of 3' dangling nucleotides in RNA helices[12] can also influence the final observed rate constants.

Supplementary Note 4. Sequences for all primers, oligonucleotides, and mRNA transcripts used in this study are presented below, written in 5' to 3' direction.

Primers:

FspI mutagenesis primer: GAGGGGTTTTTGCGCAAAGGAGGAACTATATCC

Tte mRNA cloning primers (for SiM-KARTS):

Forward:
GATCATGGATCCTAATACGACTCACTATAGGGGAACTCCTACTACAAGTTGCTAAGAGGC

Reverse: GATCATAAGCTTGCTTCCTCATCGTTCTCTGTAAACTC

Oligonucleotides:

DNA Capture Strand: GCCTCTTAGCAACTTGTAGTAGGAGTTCCAAAAAAAAAA-biotin

Cy3-Blocking Strand DNA: Cy3-GGCACAAAATTACCTCCCTTGTTTTGTTAACTGGG

LNA probe: TYE563-+GT+CAAATTT+CA+CAA+CT+C+CTTT+C, where a preceding "+" indicates a locked nucleic acid (LNA) base

RNA Anti-SD probe: Cy5-(aminohexyl-linker)-GAUCACCUCCUU

RNA Control probe: Cy5-(aminohexyl-linker)-GCAACAAGAGC

***Tte* mRNA for SiM-KARTS:**

GGGGAACUCCUACUACAAGUUGCUAAGAGGCUAUUUUUUAGUUCAAAUUACUCAUACAA
UCAUGUUAAAAUUAAUCGCAGUGAGCAACAAAAUGCUCACCUGGGUCGCAGUAACCCC
AGUUAACAAAACAAGGGAGGUAAUUUUGUGCCCAAAAAAAGAAUAAAAGAUUUAGCUG
AAAUUGCUCUUGUUGCAGCAAUUUAUUUCGCACUCACAAUUAUAUUUUCGUCCAUUUC
GUUUUUACCCGUUCAAUUUCGAAUCGGGGAAAUUACGAAAUCCAUUGUAGUAUUCAAU
AAAAAAUAUGCUAUUUCCAUGAUGAUAGGAAAUUUUUUUGCAAAUUUGUUUAGCCCAU
UUGCUGGUGCAAUGGAAUUAAUUUUUAUGCCUCUUUCGAACUUAAUAGGCUGUACAAU
UGGAUACUACAUUGGAAGACUUACUCACAAAGCGAUAGGAGCUAUAUUCAUAGCCCUU
UGGAUUGCAGCAUCAGUUGCAAUUACUUUAAAGGUUUCUGCAGGCAUACCAUUUAUUC
CGACUUUCUUAAGCGUGGGAGUAGCGGAAACUGUACUUUUGGUAACUGGAUAUUUUU
UGCUUUUCACAAUUGAAAAGAAAGGAGUUGUGAAAUUUGACAGAUAAAUAUAAAGAGA
GAAGAUUUGACAUUUACGGUUACGAAAAAAUUGACAAAGAAGUUCUAGAAUCUAUUGA
AUAUGAGUAUCCUGAAAAAAAUACUAUCGUGGAGUAUAUUACCGAUGAAUUUUCUUCU
GUUUGCCCUUGGACAGGAUUACCUGACAAUGCAAAACUUACUAUAAGGUAUAUACCCC
ACAAAAAACUUGUAGAACUUAAAUCCUUAAAAUAUUACCUUACAUCUUAUAGGAAUGUA
GGUAUAUUGCAAGAACAUGCAAUAAACAGAAUUUUAGAUGAUUUGGUGGAAUUCCUGC
AGCCAAAAUUUAUGGAAAUAAUAGGCGAAUUUCAGGAAAGAGGAGGAAUAGCUACAAG
AAUUAUAGCAAGGUAUGAAAAAGAGGAGUAUUAAACUUAAAAGGCUGCCUAAAAUUUU
GUAGGCAGCUUUUUUAUUCAUUUUAGUUUUUCUUCAAAAUGAGUUUACAGAGAACGAU
GAGGAAGCAAGCU

Tte mRNA for _in vitro_ translation:

GGGCAGUGAGCAACAAAAUGCUCACCUGGGUCGCAGUAACCCCAGUUAACAAAACAAGG
GAGGUAAUUUUGUGCCCAAAAAAAGAAUAAAAGAUUUAGCUGAAAUUGCUCUUGUUGC
AGCAAUUUAUUUCGCACUCACAAUUAUAUUUUCGUCCAUUUCGUUUUUACCCGUUCAA
UUUCGAAUCGGGGAAAUUACGAAAUCCAUUGUAGUAUUCAAUAAAAAAUAUGCUAUUU
CCAUGAUGAUAGGAAAUUUUUUUGCAAAUUUGUUUAGCCCAUUUGCUGGUGCAAUGG
AAUUAAUUUUUAUGCCUCUUUCGAACUUAAUAGGCUGUACAAUUGGAUACUACAUUGG
AAGACUUACUCACAAAGCGAUAGGAGCUAUAUUCAUAGCCCUUUGGAUUGCAGCAUCA
GUUGCAAUUACUUUAAAGGUUUCUGCAGGCAUACCAUUUAUUCCGACUUUCUUAAGCG
UGGGAGUAGCGGAAACUGUACUUUUGGUAACUGGAUAUUUUUUGCUUUUCACAAUUG
AAAAGAAAGGAGUUGUGAAAUUUGACAGAUAAAUAUAAAGAGAGAAGAUUUGACAUUU
ACGGUUACGAAAAAAUUGACAAAGAAGUUCUAGAAUCUAUUGAAUAUGAGUAUCCUGA
AAAAAAUACUAUCGUGGAGUAUAUUACCGAUGAAUUUUCUUCUGUUUGCCCUUGGACA
GGAUUACCUGACAAUGCAAAACUUACUAUAAGGUAUAUACCCCACAAAAAACUUGUAGA
ACUUAAAUCCUUAAAAUAUUACCUUACAUCUUAUAGGAAUGUAGGUAUAUUGCAAGAA
CAUGCAAUAAACAGAAUUUUAGAUGAUUUGGUGGAAUUCCUGCAGCCAAAAUUUAUGG
AAAUAAUAGGCGAAUUUCAGGAAAGAGGAGGAAUAGCUACAAGAAUUAUAGCAAGGUA
UGAAAAAGAGGAGUAUUAAACUUAAAAGGCUGCCUAAAAUUUUGUAGGCAGCUUUUUU
AUUCAUUUUAGUUUUUCUUCAAAAUGAGUUUACAGAGAACGAUGAGGAAGCAAGCU

CAT control mRNA for _in vitro_ translation:

GGGAGACCACAACGGUUUCCCUCUAGAAAUAAUUUUGUUUAACUUUAAGAAGGAGAUAU
ACAUAUGGAGAAAAAAAUCACUGGAUAUACCACCGUUGAUAUAUCCCAAUGGCAUCGU
AAAGAACAUUUUGAGGCAUUUCAGUCAGUUGCUCAAUGUACCUAUAACCAGACCGUUC
AGCUGGAUAUUACGGCCUUUUUAAAGACCGUAAAGAAAAAUAAGCACAAGUUUUAUCC
GGCCUUUAUUCACAUUCUUGCCCGCCUGAUGAAUGCUCAUCCGGAAUUCCGUAUGGC

AAUGAAAGACGGUGAGCUGGUGAUAUGGGAUAGUGUUCACCCUUGUUACACCGUUUU
CCAUGAGCAAACUGAAACGUUUUCAUCGCUCUGGAGUGAAUACCACGACGAUUUCCGG
CAGUUUCUACACAUAUAUUCGCAAGAUGUGGCGUGUUACGGUGAAAACCUGGCCUAU
UUCCCUAAAGGGUUUAUUGAGAAUAUGUUUUUCGUCUCAGCCAAUCCCUGGGUGAGU
UUCACCAGUUUUGAUUUAAACGUGGCCAAUAUGGACAACUUCUUCGCCCCCGUUUUCA
CCAUGGGCAAAUAUUAUACGCAAGGCGACAAGGUGCUGAUGCCGCUGGCGAUUCAGG
UUCAUCAUGCCGUUUGUGAUGGCUUCCAUGUCGGCAGAAUGCUUAAUGAAUUACAACA
GUACUGCGAUGAGUGGCAGGGCGGGGCGCAUCAUCAUCAUCAUCAUUAAGGAUCCGA
AUUCGAGCUCCGUCGACAAGCUUGCGGCCGCACUCGAGCACCACCACCACCACCACU
GAGAUCCGGCUGCUAACAAAGCCCGAAAGGAAGCUGAGUUGGCUGCUGCCACCGCUG
AGCAAUAACUAGCAUAACCCCUUGGGGCCUCUAAACGGGUCUUGAGGGGUUUUUUGC

Supplementary References

1. Müller, P., Schwille, P. & Weidemann, T. PyCorrFit-generic data evaluation for fluorescence correlation spectroscopy. *Bioinformatics* **30**, 2532-2533 (2014).
2. Li, G. W., Oh, E. & Weissman, J. S. The anti-Shine-Dalgarno sequence drives translational pausing and codon choice in bacteria. *Nature* **484**, 538-541 (2012).
3. Blanco, M. & Walter, N. G. Analysis of complex single-molecule FRET time trajectories. *Methods Enzymol.* **472**, 153-178 (2010).
4. Johnson-Buck, A. *et al.* Kinetic fingerprinting to identify and count single nucleic acids. *Nat. Biotechnol.* **33**, 730-732 (2015).
5. Jungmann, R. *et al.* Single-molecule kinetics and super-resolution microscopy by fluorescence imaging of transient binding on DNA origami. *Nano Lett.* **10**, 4756-4761 (2010).
6. Dupuis, N. F., Holmstrom, E. D. & Nesbitt, D. J. Single-molecule kinetics reveal cation-promoted DNA duplex formation through ordering of single-stranded helices. *Biophys. J.* **105**, 756-766 (2013).
7. Eimer, W., Williamson, J. R., Boxer, S. G. & Pecora, R. Characterization of the overall and internal dynamics of short oligonucleotides by depolarized dynamic light scattering and NMR relaxation measurements. *Biochemistry* **29**, 799-811 (1990).
8. Ouldridge, T. E., Sulc, P., Romano, F., Doye, J. P. & Louis, A. A. DNA hybridization kinetics: zippering, internal displacement and sequence dependence. *Nucleic Acids Res.* **41**, 8886-8895 (2013).
9. Schwille, P., Oehlenschlager, F. & Walter, N. G. Quantitative hybridization kinetics of DNA probes to RNA in solution followed by diffusional fluorescence correlation analysis. *Biochemistry* **35**, 10182-10193 (1996).
10. Cisse, I. I., Kim, H. & Ha, T. A rule of seven in Watson-Crick base-pairing of mismatched sequences. *Nat. Struct. Mol. Biol.* **19**, 623-627 (2012).
11. Snoussi, K. & Leroy, J. L. Imino proton exchange and base-pair kinetics in RNA duplexes. *Biochemistry* **40**, 8898-8904 (2001).
12. O'Toole, A. S., Miller, S. & Serra, M. J. Stability of 3' double nucleotide overhangs that model the 3' ends of siRNA. *RNA* **11**, 512-516 (2005).
